# Hepatotoxic pyrrolizidine alkaloids induce DNA damage response in rat liver in a 28-day feeding study

**DOI:** 10.1007/s00204-020-02779-2

**Published:** 2020-05-17

**Authors:** Johanna Ebmeyer, Josef Daniel Rasinger, Jan G. Hengstler, Dirk Schaudien, Otto Creutzenberg, Alfonso Lampen, Albert Braeuning, Stefanie Hessel-Pras

**Affiliations:** 1 German Federal Institute for Risk Assessment, Max-Dohrn-Str. 8-10, 10589 Berlin, Germany; 2 Institute of Marine Research (IMR), Postboks 1870 Nordnes, NO-5817, Bergen, Norway; 3 Leibniz Research Centre for Working Environment and Human Factors, Technical University Dortmund, Ardeystr. 67, 44139 Dortmund, Germany; 4 Fraunhofer Institute for Toxicology and Experimental Medicine ITEM, Nikolai-Fuchs-Straße 1, 30625 Hanover, Germany

**Keywords:** Pyrrolizidine alkaloids, Hepatotoxicity, Transcriptomics, DNA damage

## Abstract

Pyrrolizidine alkaloids (PA) are secondary plant metabolites that occur as food and feed contaminants. Acute and subacute PA poisoning can lead to severe liver damage in humans and animals, comprising liver pain, hepatomegaly and the development of ascites due to occlusion of the hepatic sinusoids (veno-occlusive disease). Chronic exposure to low levels of PA can induce liver cirrhosis and liver cancer. However, it is not well understood which transcriptional changes are induced by PA and whether all hepatotoxic PA, regardless of their structure, induce similar responses. Therefore, a 28-day subacute rat feeding study was performed with six structurally different PA heliotrine, echimidine, lasiocarpine, senecionine, senkirkine, and platyphylline, administered at not acutely toxic doses from 0.1 to 3.3 mg/kg body weight. This dose range is relevant for humans, since consumption of contaminated tea may result in doses of ~ 8 µg/kg in adults and cases of PA ingestion by contaminated food was reported for infants with doses up to 3 mg/kg body weight. ALT and AST were not increased in all treatment groups. Whole-genome microarray analyses revealed pronounced effects on gene expression in the high-dose treatment groups resulting in a set of 36 commonly regulated genes. However, platyphylline, the only 1,2-saturated and, therefore, presumably non-hepatotoxic PA, did not induce significant expression changes. Biological functions identified to be affected by high-dose treatments (3.3 mg/kg body weight) comprise cell-cycle regulation associated with DNA damage response. These functions were found to be affected by all analyzed 1,2-unsaturated PA.

In conclusion, 1,2-unsaturated hepatotoxic PA induced cell cycle regulation processes associated with DNA damage response. Similar effects were observed for all hepatotoxic PA. Effects were observed in a dose range inducing no histopathological alterations and no increase in liver enzymes. Therefore, transcriptomics studies identified changes in expression of genes known to be involved in response to genotoxic compounds at PA doses relevant to humans under worst case exposure scenarios.

**Electronic supplementary material:**

The online version of this article (10.1007/s00204-020-02779-2) contains supplementary material, which is available to authorized users.

## Introduction

Pyrrolizidine alkaloids (PA) are a group of secondary plant metabolites that belong to the most widely distributed natural toxins. PA can contaminate food and feed and, thus, may affect health of consumers, livestock and wildlife (Stegelmeier et al. 1999; Wiedenfeld 2011). In Germany and Europe, tea, honey and herbal spices were identified as the main sources contributing to human exposure to PA (BfR 2018; BfR 2019; Bodi et al. 2014; EFSA 2017; Mulder et al. 2018). The European Food Safety Authority and the German Federal Institute for Risk Assessment concluded that uptake of contaminated food from the European Market may pose a risk to human health, especially for people consuming high amounts of these products, and for children (BfR 2018; BfR 2019; EFSA 2017).

**Fig. 1:**
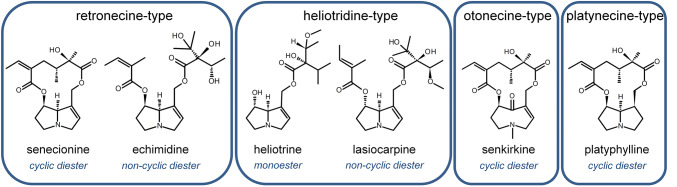
Structure of the PA representatives senecionine, echimidine, heliotrine, lasiocarpine, senkirkine and platyphylline used in this study. These six PA representatives cover the different necine structure types as well as the different degrees of esterification.

PA share a common basic structure. They consist of a 1-hydroxymethylpyrrolizidine (necine base) esterified with one or two aliphatic mono- or dicarboxylic acids, so-called necine acids. According to their necine base, they are divided into four different structure types: retronecine-, heliotridine-, otonecine- and platynecine-type PA. Furthermore, they can be grouped according to their degree of esterification into monoesters, non-cyclic diesters and cyclic diesters. Due to different combinations of different necine bases and necine acids, a wide variety of structurally different PA congeners exists. Today, several hundred PA are known; probably produced by more than 6000 plant species (Stegelmeier et al. 1999). Oxidation of the nitrogen atom at one bridgehead of the two fused pentagonal carbon rings leads to PA *N*-oxides. PA *N*-oxides show higher water solubility compared to the free bases, and, thus, the *N*-oxide type is the predominating form in plants (Wiedenfeld et al. 2008). Since PA *N*-oxides can be reduced to the free base form, it is considered that PA *N*-oxides and free PA exert comparable toxicity (Wiedenfeld et al. 2008). Figure 1 summarizes the structure of the PA representatives used in this study and assigns them to their respective structure type.

While the retronecine-, heliotridine- and otonecine-type PA are based on a 1,2-unsaturated necine base, the platynecine type lacks this double bond in 1,2-position. The 1,2-double bond is required for toxicity. Therefore, platynecine-type PA are assumed to be non-toxic. Further requirements for toxicity are described as follows: (i) at least one hydroxyl group needs to be attached to the pyrrolizidine ring via one carbon atom, (ii) at least one of this hydroxyl groups needs to be esterified, and (iii) the acid moiety needs to have a branched chain (Mattocks 1986). Toxicity is assumed to increase from monoesters to non-cyclic diesters and finally cyclic diesters with heliotridine-type PA being more toxic compared to retronecine-type PA (Merz and Schrenk 2016). It is considered that toxicity is not based on the PA parent compounds themselves but on their metabolites formed during bioactivation reactions located mainly in the liver. Hepatic metabolism is initiated by a cytochrome P450 (CYP)-mediated oxidation followed by spontaneous dehydration. Resulting pyrrolic esters are highly reactive and able to form protein and DNA adducts as well as DNA/protein cross-links which are assumed to be responsible for toxicity (Mattocks 1986; Ruan et al. 2014; Wiedenfeld et al. 2008).

Consumption of PA-contaminated food may lead to severe liver failure. Several case studies report the development of veno-occlusive disease after uptake of highly PA-contaminated wheat flour, seeds or herbal tea (Bras et al. 1954; Datta et al. 1978; Kakar et al. 2010; Mohabbat et al. 1976; Tandon et al. 1976). Furthermore, genotoxic, carcinogenic and pneumotoxic properties of PA have been described (Culvenor et al. 1969; Fu et al. 2004; Moreira et al. 2018). Some studies also point to the development of cholestasis (Hessel-Pras et al. 2020; Luckert et al. 2015). However, most of these specific effects were observed in the high-dose range. Our study aimed to identify toxic effects in a non-acute toxic dose range reflecting human exposure scenarios over a time period of 28 days to display repeated exposure and to achieve substantial responses on gene expression level in the low dose range. Furthermore, we wanted to test if structurally different PA affect different pathways or result in a similar toxicity pattern. Therefore, we chose a whole-genome transcriptomic approach in male Fischer rats treated by gavage with the PA representatives echimidine, heliotrine, lasiocarpine, senecionine, senkirkine and platyphylline covering all existing necine base structure types as well as monoesters, non-cyclic and cyclic diesters. Platyphylline is considered to be non-hepatotoxic due to the missing 1,2-double bond and was, therefore, included as a non-toxic control.

## Materials and methods

### Chemicals

Echimidine (94% purity), heliotrine (91% purity), lasiocarpine (98% purity), senecionine (99% purity) and senkirkine (98% purity) were purchased from PhytoLab (Verstenbergsgreuth, Germany). Platyphylline (95% purity) was purchased from BOC Sciences (New York, New York, USA). Senecionine was dissolved in 0.2-M hydrochloric acid. Afterwards, pH was adjusted to 6–7 using 0.2-M sodium hydroxide solution. Sodium chloride (NaCl) was added to obtain a solution containing 0.15-M NaCl. All other PA were dissolved in 0.15-M aqueous NaCl solution.

### Animal experiments

A 28-day oral toxicity study was performed with male Fischer rats (strain F-344/DuCrl) exposed to six PA, namely echimidine, heliotrine, lasiocarpine, senecionine, senkirkine and platyphylline at the Fraunhofer Institute for Toxicology and Experimental Medicine (ITEM, Hanover, Germany). All animal experiments were conducted in compliance with the regulation of the German Animal Protection Law, following the principles of Good Laboratory Practice (OECD 1998) and considering OECD Guideline 407 for repeated dose 28-day oral toxicity study in rodents (adopted guideline; October 3, 2008).

A total of 128 male Fischer rats (strain F-344/DuCrl) were purchased from Charles River Deutschland (Sulzfeld, Germany) and divided into 25 groups. Grouping was performed randomly according to the average body weight of the animals resulting in groups with the same average body weight. Each treatment group consisted of five animals. Vehicle control included 8 animals. Following delivery, the 4–5-week-old animals were allowed to acclimatize for 4 weeks; at study start (i.e., at an age of 8–9 weeks), body weights of about 200 g were determined. Animals were group-housed in type IV Makrolon® (polycarbonate) cages and maintained under conventional laboratory conditions. The temperature (22 ± 2 °C) and the relative humidity (55 ± 15%) of the animal room were monitored and recorded continuously. A 12-h light/dark cycle was used. Rats had access to commercial diet (Ssniff V1534, Ssniff-Spezialdiäten, Soest, Germany) and tap water ad libitum. Animals were treated for 28 days daily by gavage with a flexible tube. A solution of 0.15-M NaCl served as vehicle control (group 1). Groups 2–25 were treated with 0.1, 0.33, 1.0, or 3.3 mg/kg body weight (bw) of single PA. Information about animal treatment is summarized in Table 1. Doses were chosen following a two-week and 13-week NTP study with riddelliine in Fischer rats (National Institutes of Health 1993). According to the publication of Merz and Schrenk (2016), the cyclic retronecine-type diester riddelliine exhibits comparable potency like senecionine which was used in this study. A dose of 3.3 mg/kg bw was chosen as the highest dose to avoid unspecific effects resulting from general toxicity.

**Table 1:** Summary of animal treatment

Substance	Daily dose (mg/kg bw)	Number of animals
Vehicle control (0.15-M NaCl)	0	8
PA (echimidine, heliotrine, lasiocarpine, senecionine, senkirkine, or platyphylline)	0.1	5
	0.33	5
	1.0	5
	3.3	5

Throughout the study, individual body weight was recorded once a week. Additionally, animals were observed daily for clinical symptoms. One day after the last administration, animals were killed and prepared for histopathological examination of lungs and livers, blood analysis and transcriptomic analysis. Animals were killed by a lethal dose of pentobarbital. A total of approximately 3-ml heparinized blood (+ Nembutal) was collected from the *vena cava caudalis* before start of whole-body perfusion for liver preparation. Liver weights were determined.

### Histopathological examination and analysis of transaminases in blood

One slice with a maximal diameter of 7 mm of the rat liver lobes 1 and 3 was fixed in buffered formalin (10%), embedded in paraffin, sectioned, and stained with hematoxylin and eosin. Levels of the transaminases aspartate aminotransferase and alanine aminotransferase in blood were analyzed by kinetic UV test at 37 °C as described before (Bergmeyer 1983).

### Transcriptomics analysis

For transcriptomics analysis of liver, RNA was extracted using the RNeasy Mini Kit (Qiagen, Hilden, Germany) in combination with QiaShredder (Qiagen, Hilden, Germany). Thus, small pieces of the deep-frozen liver lobe 1 were homogenized under liquid nitrogen and transferred to RLT buffer (Qiagen, Hilden, Germany) containing 1% β-mercaptoethanol. Afterwards, the lysate was further homogenized using QiaShredder and the RNA was isolated using RNeasy Mini Kit according to the Manufacturer’s instructions. Concentration and purity of total RNA was measured at a Tecan M200Pro spectrophotometer using a NanoQuant Plate at wavelengths of 260 nm and 280 nm, and on an Agilent 2100 BioAnalyzer (Agilent, Santa Clara, California, USA). Only high-quality RNA with RNA integrity numbers (RIN) higher than 9 were used for microarray analysis. Microarray analysis was performed at Eurofins (Aarhus, Denmark) using Affymetrix GeneChip Rat ClariomD arrays (Thermo Fisher Scientific, Waltham, Massachusetts, USA). The raw data of this publication have been deposited in NCBI's Gene Expression Omnibus (Edgar et al. 2002) and are accessible through GEO Series accession number GSE149678.

### Verification of microarray results by qPCR

Extracted RNA was analyzed by quantitative real-time PCR (qPCR) for the expression of the following genes to validate microarray results: *Abcb1b, Aldh1a1, Ckap2, Fas, Clec4f, Csf1r* and *Inmt*. The first 4 genes were found to be significantly (q < 0.05) upregulated in microarray analysis. The last three genes were significantly (*q* < 0.05) downregulated. The genes were randomly chosen among the genes that were dysregulated by at least 3 out of 5 toxic PA. *Gapdh* and *Actb* were used as housekeeping genes. Extraction of RNA was performed as described for transcriptomics analysis (see Sect. “Transcriptomics analysis”). Afterwards, cDNA synthesis was conducted with 1000-ng RNA using the High Capacity cDNA Reverse Transcription Kit according to the manufacturer’s instructions (Applied Biosystems, Foster City, California, USA). qPCR was performed using Maxima SYBR Green/ROX qPCR Master Mix (Thermo Fisher Scientific, Waltham, Massachusetts, USA) with 1 µl of cDNA and 300 pmol of the respective forward and reverse primers on a Stratagene Mx3005P instrument (Agilent Technologies, Santa Clara, California, USA). Gene names, gene abbreviations, and used primer sequences are summarized in Table 2. All primers were designed using Primer3 software (Rozen and Skaletsky 2000). For thermal cycling procedure, an initial denaturation and activation of the hot start Taq polymerase at 95 °C for 15 min was followed by 40 cycles consisting of denaturation (95 °C, 15 s) and annealing and elongation (60 °C, 1 min). Finally, final elongation was performed for 15 min at 60 °C. Additionally, a dissociation curve step was added.

**Table 2:** Primer sequences used in this study

Gene^1^	Sequence (5′–3′)	
	Forward primer	Reverse primer
*Abcb1b*	TGGCCATGTACGCCTACTATTACA	AAAACCGGCTGAAAATGTCGTT
*Actb*	TGTGTTGTCCCTGTATGCCT	AGCGCGTAACCCTCATAGAT
*Aldh1a1*	GATGCCGACTTGGACATTGC	GACGCAGCATTGGCCTTGAT
*Ckap2*	TAATGCCAGGCTGACAGGAA	GCTGTGGTATCTTTGGGCTG
*Clec4f*	ATGAAGGAGGCGGAACTGAA	ATGGGAGCAGCTGACTTAGG
*Csf1r*	CAAACTCCACCTGAACCGTG	TATCGCAGGGTGAGCTCAAA
*Fas*	ACATCTGGAGAACTGCCGAA	CATCGATCTTGCTTTCCGGG
*Gapdh*	CCGTGGGGCAGCCCAGAA	GCCCCAGCATCAAAGGTGGAGGA
*Inmt*	ACTAGAATGGCAGGCAAGGT	GCTGTAGAAGGTGGTCAGGT

^1^Gene names: *Abcb1* ATP-binding cassette subfamily B member 1, *Actb* actin beta, *Aldh1a1* aldehyde dehydrogenase 1 family member A1,* Ckap2* cytoskeleton-associated protein 2,* Clec4* C-type lectin domain family 4,* Csf1r* colony stimulating factor 1 receptor,* Fas* fas cell surface death receptor,* Gapdh* glyceraldehyde-3-phosphate dehydrogenase,* Inmt* indolethylamine N-methyltransferase

### Statistical and bioinformatic data analysis

For statistical analysis, microarray data were normalized using the robust multi-array-average (RMA) function of the limma (linear models for microarray and RNA-Seq data) package (Smyth et al. 2019, page 104) in R (R Core Team 2017; Ritchie et al. 2015) to assure that any batch effects between arrays are being removed. Subsequently, the dataset was filtered and only transcripts were kept for which annotations were available. The reduced dataset comprising 26,480 probe sets was analyzed further using Qlucore Omics Explorer (version 3.3, Qlucore, Lund, Sweden). The short technical note on the use of Qlucore for omics data mining gives a short summary of how the analysis workflow employed in the present study has been used to reduce large number of altered genes to a few robust discriminative features (Rasinger and Lie). After a correction for batch-dependent differences of microarray chips, a comparison of all high-dose groups of single PA was performed using one-way analysis of variance (ANOVA). Fold changes for significantly dysregulated genes (*q* < 0.05) were determined using two-group comparison tests against vehicle control. The *q*-values are Benjamini–Hochberg-corrected p-values as outputted by Qlucore (Benjamini and Hochberg 1995) to control the false discovery rate. In addition, rank regressions were performed across all doses for each individual PA.

Following statistical analysis, data was subjected to Ingenuity Pathway Analysis (IPA, version 48,207,413, Qiagen Bioinformatics, Redwood City, California, USA). Comparison analyses to predict affected canonical pathways, diseases and functions and upstream regulators were performed.

Data regarding body weight, relative liver weight, levels of transaminases were recorded and analyzed by one-way ANOVA followed by Dunnett’s post hoc test using the PROVANTIS system (version 8.4.3.1). Relative gene expression as analyzed by qPCR was statistically analyzed by one-way ANOVA followed by Dunnett’s post hoc test using SigmaPlot software.

## Results

### Body weights, organ weights and transaminases in blood

No differences in body weight development of treated animals compared to the vehicle control were observed. Individual data are summarized in supplementary data Figure S1. Absolute liver weights did also not show relevant differences between vehicle control and treatment groups. The high-dose senkirkine treatment group showed a slightly but significantly increased relative liver weight (111% of vehicle control, *p* < 0.05) (Supplementary data, Fig. S2).

The levels of the hepatic enzymes aspartate aminotransferase (AST) and alanine aminotransferase (ALT) were determined in blood to identify hepatotoxicity. All groups did not show statistically significant (*p* < 0.05) changes in neither ALT nor AST levels, except the high-dose group of lasiocarpine (3.3 mg/kg bw) which showed a statistically significant (*p* < 0.05) increase of ALT from 85.8 ± 22.5 U/l in control animals to 160.5 ± 19.4 U/l (Supplementary data Fig. S3). However, no morphological changes were detected in this group during histopathological examination (see “Histopathology”).

### Histopathology

The livers of all animals were examined histopathologically. A (multi)focal random mixed inflammatory cell infiltration as well as (multi)focal periportal mononuclear cell infiltration were seen to a very slight extent in up to two animals per group in most groups (Supplementary data Fig. S4). Within the senecionine high-dose group (3.3 mg/kg bw), four animals showed a (multi)focal random mixed inflammatory cell infiltration (3/5 very slight and 1/5 slight). However, these lesions occur occasionally in rats and are considered to be unrelated to the treatment.

Taken together, results of biochemical analysis showed only weak effects for lasiocarpine in the highest dose group by inducing a twofold increase in ALT activity. This suggests that the chosen dose range represents doses in the borderline range for classic toxicity and, thus, may be in the range of or below the no-observed adverse effect levels (NOAEL). Therefore, it is assumed that the changes in the following gene expression analysis are PA-specific effects instead of unspecific effects resulting from massive injury of the whole organism.

### Whole-genome microarray analysis

**Fig. 2:**
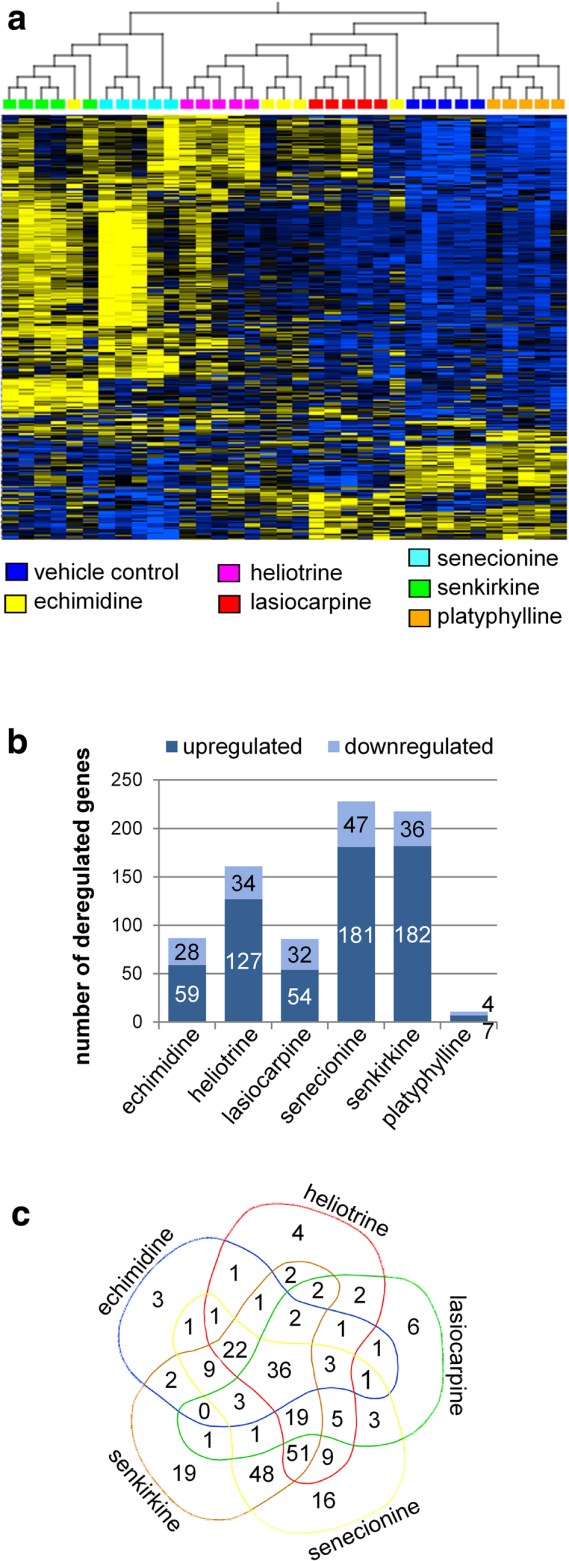
Comparison of dysregulated probe sets after oral treatment of male Fischer rats by gavage for 28 days with six different PA. Results for the high dose groups (3.3 mg/kg bw) are presented. **a** Clustering of gene expression changes in the liver following exposure to PA. Gene expression profiles were analyzed using Affymetrix microarrays. Rats were exposed to either 3.3 mg/kg bw echimidine (yellow), heliotrine (pink), lasiocarpine (red), senecionine (light blue), senkirkine (green), platyphylline (orange) or a solution of 0.15 M NaCl serving as vehicle control (dark blue). Each column represents a single animal. Each row shows a specific transcript. Microarray data were normalized, batch corrected and statistically analyzed using Qlucore Omics Explorer. A total of 284 probe sets were identified to be significantly differentially regulated (*q* < 0.05, ANOVA). Individual PA exposure effects and fold changes were determined by performing two-group comparison test versus the vehicle control. **b** The bar chart shows the average numbers of up- and downregulated probe sets for each treatment group. **c** The Venn diagram shows the average number of dysregulated probe sets for the different treatment groups (high dose groups with 3.3 mg/kg bw only) and their overlap. The Venn diagram was generated using https://bioinformatics.psb.ugent.be/webtools/Venn/

A whole-genome microarray analysis was performed to identify PA-affected pathways in dependence of the PA structure type. For statistical evaluation, the whole dataset including 70,580 probe sets was reduced to 26,480 annotated probe sets and an ANOVA between all high-dose groups and vehicle control was performed. This resulted in 284 significantly altered (*q* < 0.05) probe sets (see Supplementary data, Table S1). Hierarchical clustering of these transcripts according to the PA exposure groups is shown in Fig. 2a.

**Fig. 3:**
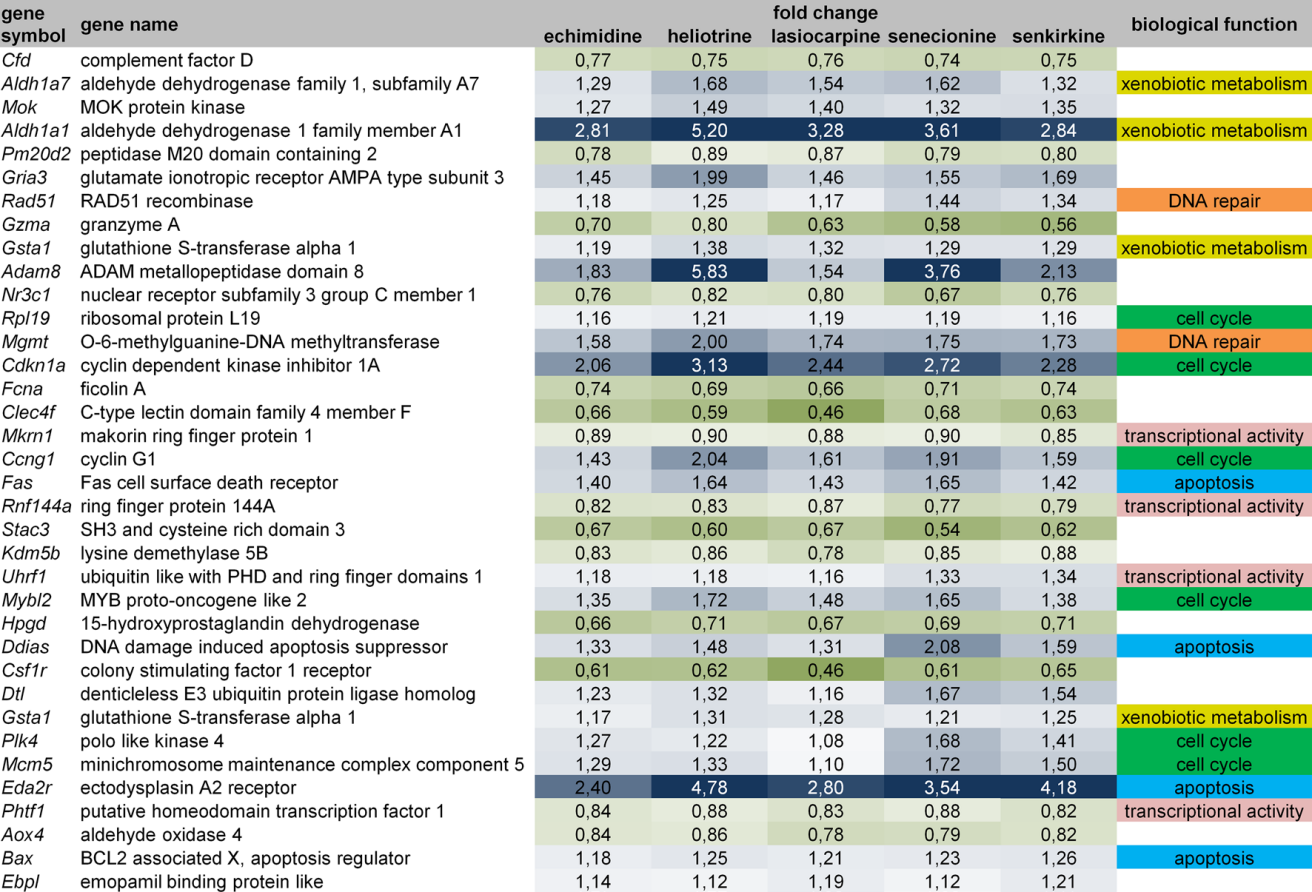
Fold change and classification of biological function of 36 probe sets corresponding to 35 individual genes that are commonly dysregulated by all 5 toxic PA according to microarray data analysis of livers of male Fischer rats orally treated daily for 28 days with 3.3 mg/kg bw of the respective PA

Platyphylline as a non-hepatotoxic PA showed a gene expression pattern very similar to the vehicle control up to the highest tested dose of 3.3 mg/kg bw. Senkirkine showed the strongest alterations compared to the vehicle control, followed by senecionine. Heliotrine and lasiocarpine showed alterations of moderate magnitude between platyphylline and senecionine/senkirkine. Echimidine was the treatment group with the highest variability in the magnitude of alterations between the different individuals. To identify the direction of regulation, significantly differentially regulated (*q* < 0.05, ANOVA) probe sets were analyzed further performing two-group comparisons of each PA against the vehicle control. The number of up- and downregulated probe sets is shown in Fig. 2b. Most of the dysregulated genes were upregulated. Again, senecionine and senkirkine showed the highest numbers of significantly dysregulated probe sets. Platyphylline, a PA representative assumed to be non-hepatotoxic, provoked significant regulation of only 11 probe sets in total. The Venn diagram in Fig. 2c shows the overlap of similar dysregulated probe sets for the different PA. In total, a signature of 36 dysregulated probe sets corresponding to 35 individual genes was identified to be dysregulated by all hepatotoxic PA. This gene signature is summarized in Fig. 3. The genes regulated by individual PA are summarized in Table S2 in the supplemental material.

This list includes a high number of genes associated with DNA damage response; cell cycle control/integrity, or apoptosis: Rad51 recombinase (*Rad51*) plays an important role during homologous recombination and is upregulated. O-6-methylguanine-DNA methyltransferase (*Mgmt*) is involved in the repair of methylated DNA and is also upregulated. Cyclin-dependent kinase inhibitor 1A (*Cdkn1a*) and Cyclin G1 (*Ccng1*) function as regulators of the cell cycle. *Cdkn1a* regulates cell cycle progression and leads to cell cycle G1 phase arrest. *Ccgn1* is associated with DNA damage-induced G2/M phase arrest. Both genes are regulated by the tumor suppressor protein p53 and were upregulated in this study. Upregulation of these genes suggests that PA may provoke DNA damage and, thus, induce expression of genes important for DNA repair and subsequent cell cycle control. The transcript for DNA damage-induced apoptosis suppressor *(Ddias*) was found to be upregulated; this may indicate a possible counter-regulation elicited to protect against DNA damage-induced cell death*.* MYB proto-oncogene like 2 (*Mybl2*) is a transcription factor that regulates cell cycle progression. Deregulation of *Mybl2* expression is associated with cancer initiation and progression (Musa et al. 2017). Polo-like kinase 4 (*Plk4*) mediates duplication of centrioles during cell cycle and was also upregulated in this study. Minichromosome maintenance complex component 5 (*Mcm5*) is important for DNA replication during G1 and S phases of the cell cycle and was identified to be upregulated. Ribosomal protein L19 (*Rpl19*) is part of the 60S subunit of the ribosome and, thus, is essential for protein synthesis. *Rpl19* was also among the upregulated common dysregulated genes. Upregulation of these three genes may point to replacement proliferation as a consequence of cell death resulting from DNA damage. Induction of cell death is furthermore emphasized by upregulation of genes related to cell death: the Fas cell surface death receptor (*Fas*) is involved in initiation of extrinsic apoptosis. An induction of apoptosis was also reported to be mediated by the ectodysplasin A2 receptor (*Eda2*r) (Sinha and Chaudhary 2004). BCL2-associated X (*Bax*) protein is another pro-apoptotic factor. Furthermore, several genes associated with transcriptional activity were identified as commonly dysregulated genes: ring finger proteins makorin ring finger protein 1 (*Mkrn1*) and ring finger protein 144A (*Rnf144a*) were downregulated, and ubiquitin like with PHD and ring finger domains 1 (*Uhrf1*) was upregulated. These three genes are associated with a novel class of zinc finger proteins that may act as transcriptional regulators. Putative homeodomain transcription factor 1 (*Phtrf1*) was downregulated. Finally, genes related to xenobiotic metabolism showed an altered expression upon treatment with PA: aldehyde dehydrogenase family 1 member A7 (*Aldh1a7*), aldehyde dehydrogenase 1 family member A1 (*Aldh1a1*) and glutathione *S*-transferase alpha 1 (*Gsta1*) encode enzymes for xenobiotic metabolism and were all upregulated in this study. A total of 48 genes were individually regulated by only one single PA (listed in Supplementary Table S2). While the low number of individual genes per PA does not allow concluding on specific mechanisms activated by only one of the test compounds, it is obvious that several genes are related to cell cycle, DNA repair or genotoxic responses, for example cyclin D1 (*Ccnd1*)*,* platelet-derived growth factor C (*Pdgfc*)*,* KiSS-1 metastasis suppressor (*Kiss1*)*,* CD244 molecule (*Cd244*)*,* caspase 12 (*Casp12*)*,* or non-homologous end-joining factor 1 (*Nhej1*), respectively.

**Fig. 4:**
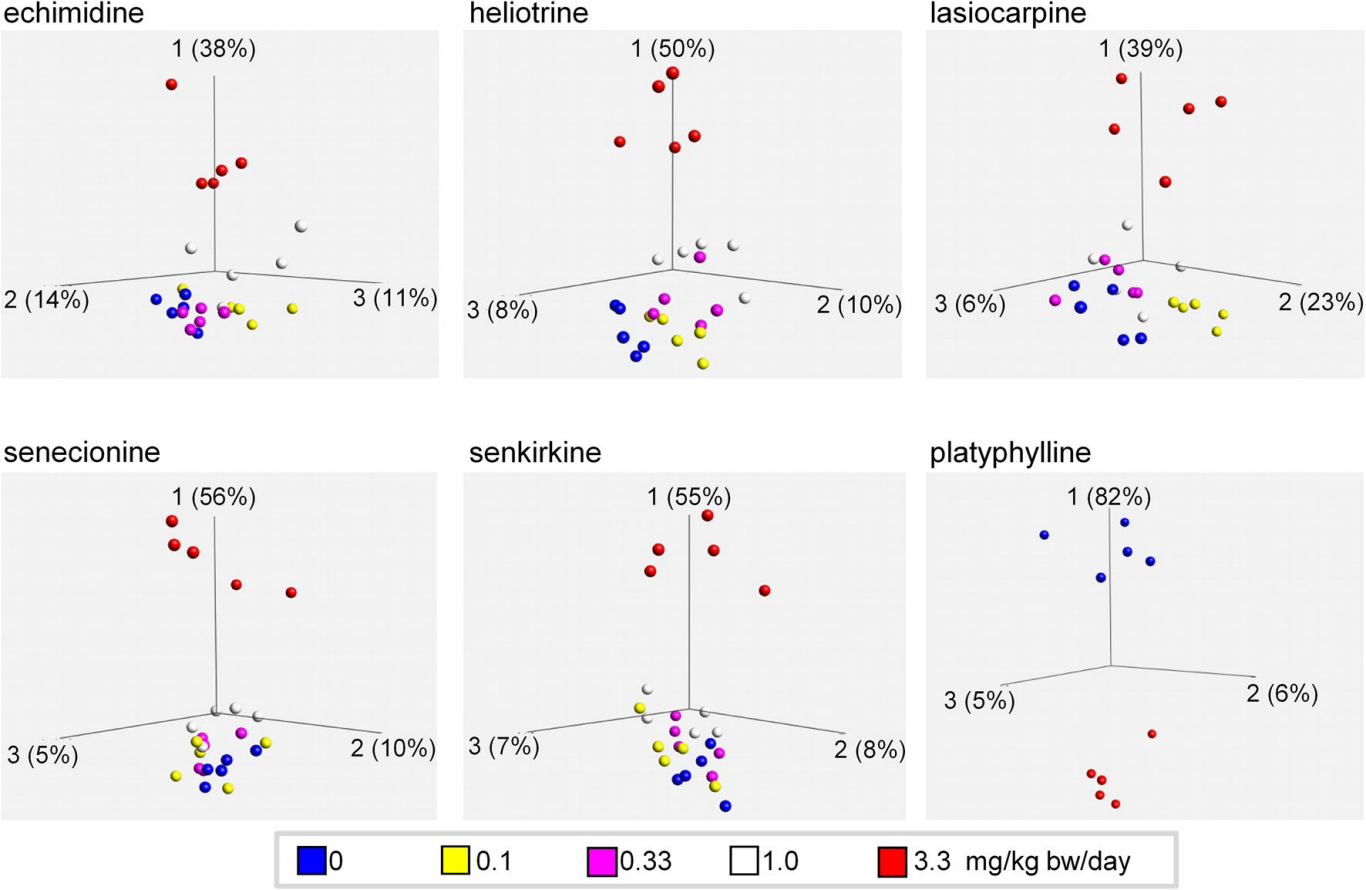
Dose dependence of gene regulation for the different treatment groups. For each PA, all tested doses were analyzed by one-way ANOVA. Differentially regulated genes (*q *< 0.05) were subjected to principal component analyses (PCA). Differences in dose-related effects are shown in the PCA plots with each individual animal being represented by a single dot

Figure 4 depicts the dose dependence of significantly deregulated (*q* < 0.05) transcripts for each PA in a principal component analysis (PCA) plot. The PCA shows that the highest dose (3.3 mg/kg bw) differs clearly from vehicle control among the main discriminate axis (PC1). A tendency of gene expression alterations compared to vehicle control was also observed for the second highest dose (1.0 mg/kg bw). These results suggest that 3.3 mg/kg bw is a dose resulting in clear effects on the transcriptome of the liver, and that 1.0 mg/kg bw was in the borderline dose range near the NOEL for transcriptomic changes.

In case of platyphylline exposure, only the highest dose was analyzed. The PCA analysis of this group, therefore, displays a more pronounced discrimination along PC1, but of opposite direction compared to the other PA. Since unlike for the other PA no lower doses were analyzed, no conclusions concerning dose response can be drawn for this PA.

### Verification of microarray data

The expression levels of 4 upregulated and 3 downregulated genes were additionally assessed by qPCR. The transcripts for qPCR validation were randomly chosen from the set of significantly dysregulated genes (*q* < 0.05) which were directly affected by at least 3 of all toxic PA in the highest-dose group. The gene expression pattern as analyzed by qPCR was similar to that of the microarray assay (see Supplementary data Fig. S5), thus verifying the microarray data.

### Ingenuity Pathway Analysis

**Fig. 5:**
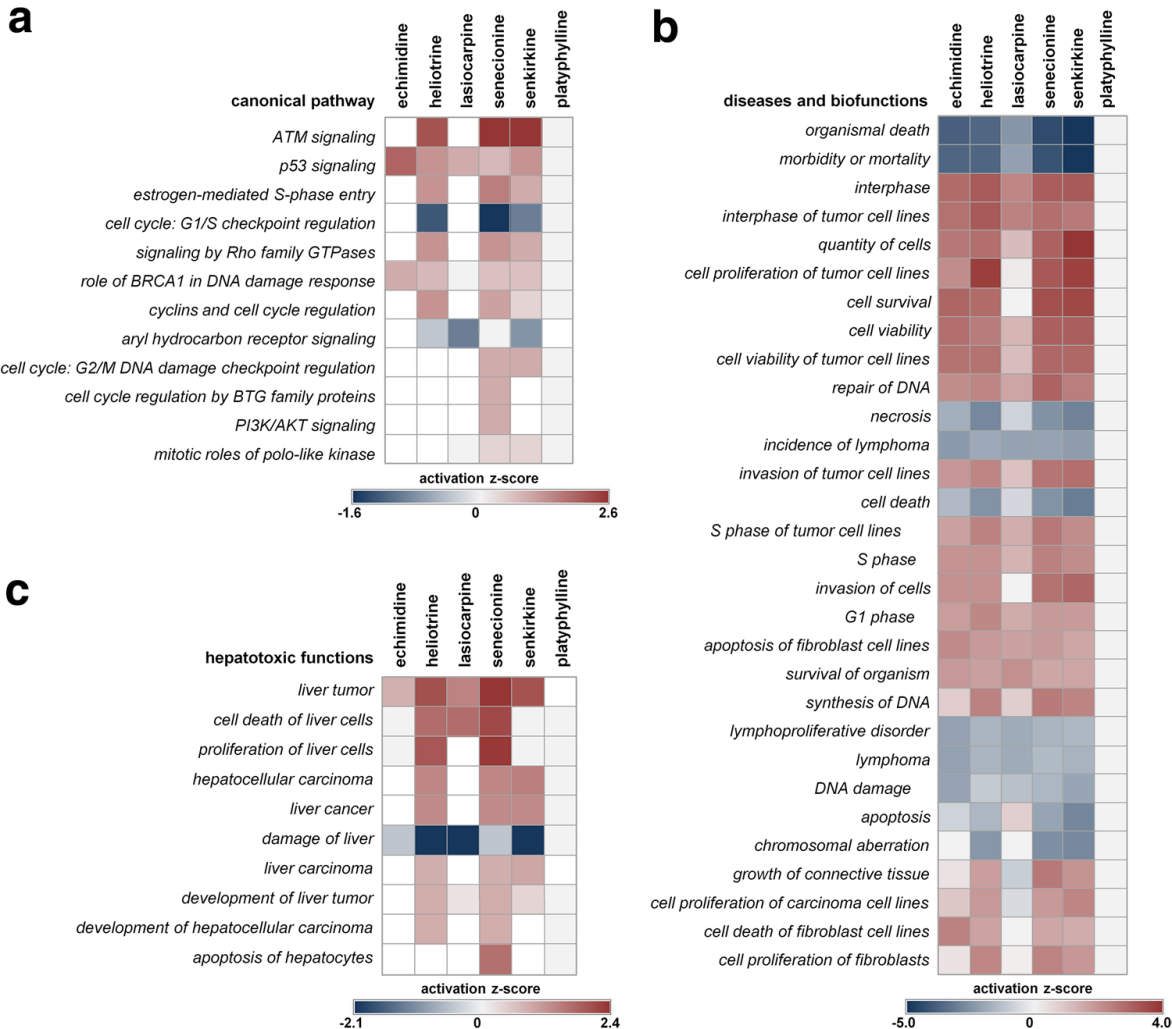
Biological functions and pathways predicted to be activated or inactivated by PA according to Ingenuity Pathway Analysis (IPA). A set of 284 significantly (*q* < 0.05) dysregulated rat liver transcripts was analyzed with IPA. Canonical pathways (**a**), diseases and biofunctions (**b**) and hepatotoxic functions (**c**) are displayed. The heatmaps show activation z-scores for the respective canonical pathways. For diseases and biofunctions, the 30 diseases and functions with the highest positive or the lowest negative activation z-scores are shown. Hepatotoxic functions were identified by IPA analysis for activation/inactivation of diseases and functions, filtered for hepatotoxicity. A complete list of affected functions is presented in the supplemental material

IPA analysis was based on a subset of 284 significantly (*q* < 0.05, ANOVA) dysregulated probe sets. For identification of a dose-dependent up- or downregulation of the transcripts, the correlation coefficient of a rank regression performed across all dose levels was uploaded to IPA. An IPA comparison analysis between all PA predicted affected canonical pathways, diseases and functions, and upstream regulators for the toxic PA echimidine, heliotrine, lasiocarpine, senecionine and senkirkine (Fig. 5). Platyphylline as a non-toxic PA representative showed no predicted activation or inhibition of any of the analyzed functions. The canonical pathway analysis predominantly predicted interaction with pathways related to DNA damage and cell cycle control/integrity (Fig. 5a).

IPA analysis found that ataxia telangiectasia mutated (ATM) signaling and tumor suppressor protein p53 signaling show the highest activation z-score. ATM signaling was predicted to be activated by heliotrine, senecionine and senkirkine; p53 signaling was predicted to be activated by all PA except platyphylline. According to the IPA knowledgebase (QIAGEN Inc., https://www.qiagenbioinformatics.com/products/ingenuity-pathway-analysis), both of these pathways are involved in initiation of DNA repair and able to lead to cell cycle arrest in the case of massive DNA damage. P53 furthermore regulates as a transcription factor the expression of several genes. Additionally, estrogen-mediated S-phase entry (activated by heliotrine, senkirkine and senecionine), G1/S checkpoint regulation (inactivated by heliotrine, senkirkine and senecionine), cyclins and cell cycle regulation (activated by heliotrine, senkirkine and senecionine), G2/M DNA damage checkpoint regulation (activated by senkirkine and senecionine), cell cycle regulation by BTG family proteins (activated by senkirkine), and mitotic roles of polo-like kinase (activated by senkirkine and senecionine) were predicted to be regulated. All of these pathways are related to cell cycle regulation. PI3K/AKT signaling was predicted to be activated by senkirkine. It regulates cell proliferation and cell death and is associated with cancer. Signaling by Rho family GTPases was predicted to be activated by heliotrine, senkirkine and senecionine. Rho family GTPases are important for regulating signal transduction and, thus, involved in several cellular processes. Finally, aryl hydrocarbon receptor signaling was predicted to be inactivated. The aryl hydrocarbon receptor is a transcription factor which regulates the expression of several genes, e.g., relevant for xenobiotic metabolism, cell proliferation and, thus, cell cycle regulation.

Most predictions in the area of diseases and functions are associated with cell viability/cell death/cell cycle, DNA damage or carcinogenesis (Fig. 5b). These predictions point to a genotoxic effect accompanied by replacement proliferation. Most of the predicted diseases and functions were affected by all PA except platyphylline. Platyphylline shows no impairment of any disease or biofunction.

Limiting the diseases and biofunctions predictions to hepatotoxicity only, resulted in predictions of functions related to liver cancer (Fig. 5c), such as stimulation of formation of liver tumor, hepatocellular carcinoma, liver cancer and liver carcinoma. Again, for platyphylline, no activation or inactivation of any hepatotoxic function was predicted.

**Fig. 6:**
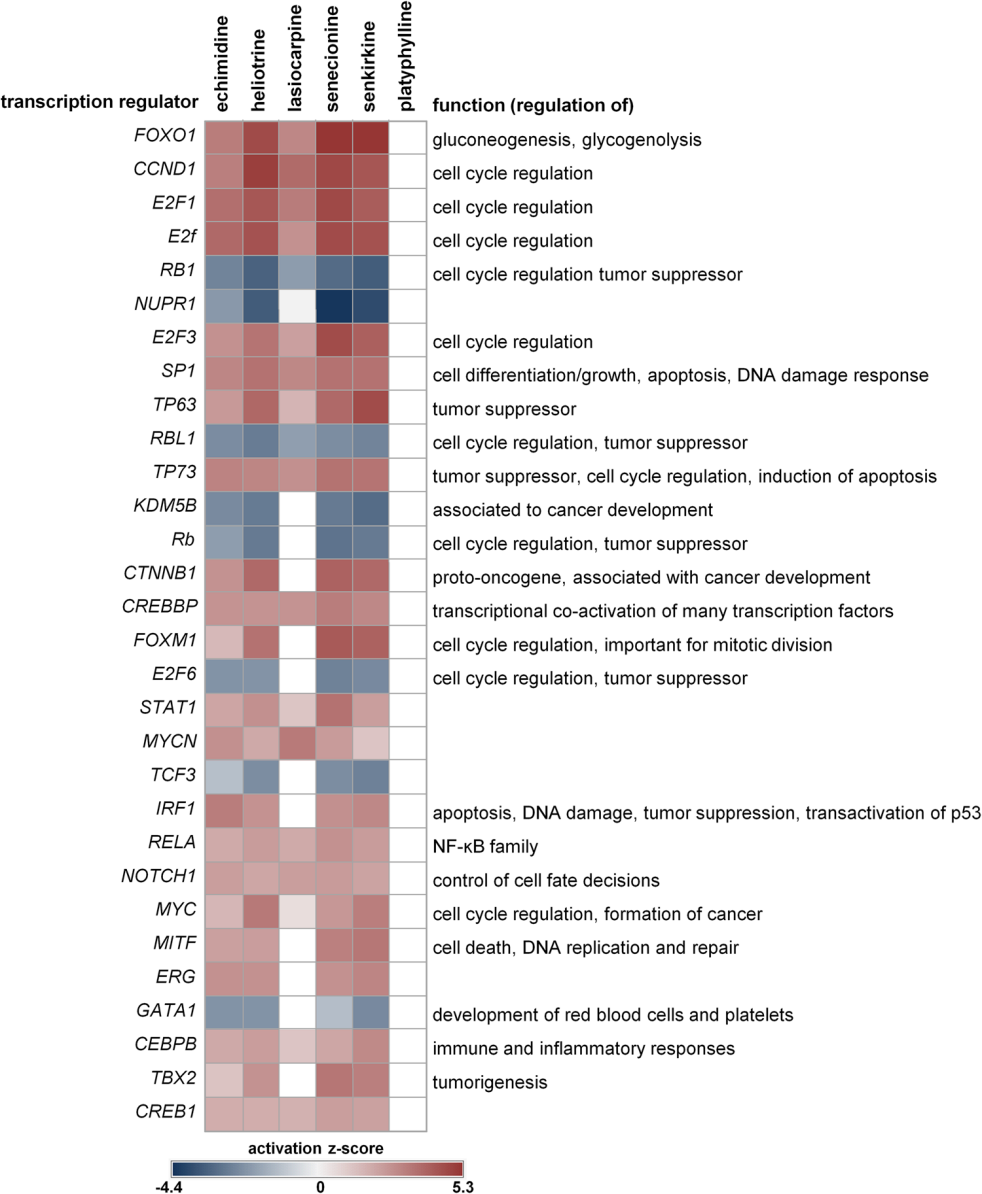
Transcription regulators predicted to be activated/inactivated by Ingenuity Pathway Analysis (IPA). A set of 284 significantly (*q* < 0.05) dysregulated rat liver transcripts was analyzed using IPA. Activation/inactivation of upstream regulators, filtered for transcription regulators, is displayed without using cutoffs. The heatmap shows the activation z-score for the respective regulator. The first 30 regulators with the highest positive or the lowest negative activation z-score are shown

IPA upstream analysis filtered for transcription regulators identified several regulators associated with cell cycle control (Fig. 6). Cyclin D1 (Ccnd1) is involved in G1/S transition during cell cycle control and was activated by all analyzed PA, except platyphylline. Ccnd1 interacts with retinoblastoma protein (Rb/Rb1). Rb/Rb1 inhibits cell cycle progression and functions as a tumor suppressor. Rb/Rb1 was downregulated on mRNA level by all PA (except platyphylline) pointing again to proliferation and possibly a promotion of cancer development. Rb/Rb1 binds also to transcription factors of the E2f family leading to its inactivation. Transcription factors of the E2f family are also important for cell cycle progression. E2f1, E2f and E2f3 were predicted to be activated by all analyzed toxic PA. E2f6 was predicted to be inactivated by all PA, except lasiocarpine and platyphylline. Further activated transcription regulators, such as β-catenin, Foxm1, Irf1, Myc, Mitf, Tbx2 with a somewhat lower z-score did also point to cell cycle regulation, DNA damage and cancer development.

## Discussion

PA belong to the most widely distributed natural toxins known. However, there is still no in vivo study that includes human-relevant doses on the one hand and structure-dependent hepatotoxicity on the other. Thus, our transcriptomic study aimed to investigate pathways affected by PA in a subacute animal study using exposure scenarios relevant for highly exposed humans. It was of particular interest whether the different PA show the same toxicity pattern or result in activation/inactivation of specific, individual pathways. Male Fischer rats were treated daily for 28 days orally with 6 structurally different PA and gene expression in the livers was analyzed using whole-genome microarrays. A daily dose of 3.3 mg/kg bw was administered as the highest dose to exclude unspecific effects resulting from general toxicity. The amount of this maximum dose results from a two-week and 13-week NTP study with the cyclic retronecine type diester riddelliine in Fischer rats (NTP 2003). According to the publication of Merz and Schrenk (2016) riddelliine exhibits comparable potency like senecionine which was used in our study. We decided to choose senecionine as representative of aforementioned PA group due to a better comparison to an existing set of in vitro and in vivo data (Hessel-Pras et al. 2020; Hessel et al. 2014; Luckert et al. 2015; Waizenegger et al. 2018). For comparing the highest dose used in our study to human exposure, human intake was estimated. Recently, a highly contaminated tea sample from the German retail market was found to contain 161 µg PA per tea bag (Stiftung Warentest 2017). Excluding PA uptake from other sources, such as herbal spices or honey, an average 60-kg adult who drinks 3 cups of this tea, would be exposed to 8-µg PA /kg bw. Therefore, the highest dose of 3.3 mg/kg bw used in the present study is about 400 times higher compared to the dose taken up by a human consuming 3 cups of the aforementioned contaminated tea; while, the lowest tested dose of 0.1 mg/kg bw is approximately 12.5-fold higher. A case report of infants describes that severe veno-occlusive disease was developed after ingestion of PA-contaminated food; the estimated daily PA uptake calculated from this report ranged from 0.8 to 3 mg/kg bw (Fox et al. 1978). The selected doses in this study, thus, represent a dose range that may be reached by human in worst case exposure scenarios. It should be noted that using higher doses of the compounds most probably would have provoked more pronounced transcriptional alterations. Nonetheless, this might have led to the masking of PA-specific effects by transcriptional signatures related to unspecific processes related to cell death and major organ damage. Therefore, the dosing was selected as described above, to allow for the detection of early transcriptional changes induced by PA.

Bioinformatic analysis identified a gene signature related to DNA damage, DNA repair, replacement proliferation/impairment of cell cycle control. Also, the IPA analysis of diseases and functions, points to DNA repair, cell death and replacement proliferation. Upstream analysis filtered for transcription regulators identified additionally regulators that affect gene expression relevant for cell cycle progression/regulation, DNA damage response and cancer formation. Most of the identified dysregulated genes were affected by all 5 or at least 4 out of 5 toxic PA, indicating a similar mode of action. The genotoxic and carcinogenic potential of PA was already described in the literature. Merz and Schrenk (2016) furthermore list an overview about several studies analyzing DNA damage, chromosomal damage and mutagenic effects induced by different PA. However, our study is not based on a classic 2-year carcinogenicity study, but on a transcriptomics approach with comparably low doses of PA for a shorter period of 28 days. The results show that this approach identifies several dysregulated genes that play a role in response to DNA damage and carcinogenesis at dose levels that may occur in humans in worst case scenarios. Recently, a toxicogenomics directory of 162 rat hepatotoxicants has been established that also contains a list of genes most frequently dysregulated by genotoxic and non-genotoxic rat carcinogens (Grinberg et al. 2018). These genes (Table S5 in Grinberg et al. 2018) show a high degree of overlap with the 35 genes shown to be deregulated by all 5 PA in the present study (Fig. 3). Among genes in the overlap of the present study and the consensus signature of Grinberg et al (2018) are *Aldh1a1*, *Cdkn1a*, *Nr3c1*, *ERpl19*, *Mgmt*, *Fna*, *Clec4f*, *Ccng1*, and *Mybl2*. No PA-induced effects were observed in the histopathological analysis; whereas, the transcriptomic approach showed clear alterations for the high-dose (3.3 mg/kg bw) PA groups. The second highest dose (1.0 mg/kg bw) seemed to be in a borderline dose range where transcriptomics effects begin to be induced. Therefore, the results point to a higher sensitivity of a transcriptomics approach in comparison to conventional toxicity studies.

In our study, senecionine and senkirkine, both cyclic diesters of the retronecine or otonecine type, respectively, showed the strongest alterations compared to the vehicle control followed by heliotrine (monoester of the heliotridine type) and lasiocarpine (non-cyclic diester of the heliotridine type). Echimidine (non-cyclic diester of the retronecine type) fluctuated in the range of heliotrine and lasiocarpine. Senecionine was identified before to belong to the most potent PA. Waizenegger et al. (2018) compared the cytotoxic and apoptotic potential of the four PA echimidine, heliotrine, senecionine and senkirkine in vitro in 24-h and 14-day exposure scenarios. Senecionine showed the strongest effects followed by echimidine. Heliotrine and senkirkine showed much weaker effects. Louisse et al. (2019) investigated the genotoxic potential of 37 structurally different PA by analyzing the phosphorylation of the histone H2AX. According to their results, senecionine, lasiocarpine and echimidine show the highest toxic potential, followed by senkirkine and finally heliotrine. Merz and Schrenk (2016) suggested the introduction of relative potency factors for PA risk assessment. Their relative interim potency factors (iRPF) are based on the combined genotoxic potency in Drosophila, cytotoxic potency in vitro and acute toxicity in adult rodents (i.p./i.v. injection). According to Merz and Schrenk, the PA senecionine, senkirkine and lasiocarpine belong to the most potent PA (iRPFs of 1.0), followed by heliotrine (iRPF 0.3) and echimidine (iRPF 0.1). The observations made in our study are very much in line with the suggested iRPFs. Some differences were observed for lasiocarpine, which showed a lower number of dysregulated genes compared to senecionine, senkirkine, echimidine and heliotrine. Compared to the results of Louisse et al. (2019), our results show a higher toxic potential for senkirkine and a lower toxic potential for echimidine and lasiocarpine. However, differences in potency can be due to the different test systems or exposure routes in the different studies. For the strong hepatotoxicity of PA, bioavailability and toxicokinetic properties most likely play a key role. Thus, our study compares for the first time the induction of hepatotoxicity by six structurally different PA after an oral exposure representing the real exposure of humans. A different intestinal first pass effect and consequently different levels of liver toxicity can be assumed due to a different passage rate over the intestinal barrier which was shown in vitro for four structurally different PA (Hessel et al. 2014). In Caco-2 cells, echimidine exhibited the lowest passage rate over the intestinal monolayer due to an ABCB1-driven efflux. Due to structural similarities between echimidine and lasiocarpine (both open-chain diester), the reduction of oral bioavailability by active efflux mechanisms can be hypothesized.

The analyzed gene signature points to DNA-damaging properties accompanied by replacement proliferation and cancer development. All analyzed hepatotoxic PA induced similar pathways. The effect strength depended on the respective PA structure type. Senecionine and senkirkine, both cyclic diesters of the retronecine and otonecine type, respectively, showed the strongest effect, followed by the non-cyclic diester of the retronecine type, echimidine, and the monoester of the heliotridine type, heliotrine. Lasiocarpine, a non-cyclic diester of the heliotridine type, induced comparably weak effects. Platyphylline, a PA representative assumed to be non-hepatotoxic, induced no relevant gene expression alterations, as expected. The detection of the DNA-damaging properties without observing histopathological changes (dose 1.0 mg PA per kg bw) and treatment duration far below classic carcinogenicity studies suggests that transcriptomic approaches may be a helpful tool to investigate the genotoxic potential of possible toxins. While omics data represent promising approaches for the early, sensitive detection of certain toxic effects which become histopathologically detectable much later, there are still several obstacles which impede the use of omics data in a regulatory context. This includes, for example, harmonization of technology platforms and data analysis workflows, as well as the definition of adversity (Marx-Stoelting et al. 2015). Future research is, thus, still needed to facilitate the implementation of omics data in regulatory toxicology.

## Electronic supplementary material

Below is the link to the electronic supplementary material.

Supplementary file1 (PDF 2092 kb)

## Abbreviations

ALT: Alanine aminotransferase
ANOVA: Analysis of variance
AST: Aspartate aminotransferase
bw: Body weight
CYP: Cytochrome P450 monooxygenase(s)
IPA: Ingenuity pathway analysis
qPCR: Quantitative real-time PCR
PA: Pyrrolizidine alkaloid(s)
PCA: Principal component analysis
