# Supplementary material for: Hepatotoxic pyrrolizidine alkaloids induce DNA damage response in rat liver in a 28-day feeding study
Source: Arch Toxicol. 2020 May 17;94(5):1739–51. doi: 10.1007/s00204-020-02779-2 (PMC7261731; doi:10.1007/s00204-020-02779-2)
Supplement: Supplementary file 1 — Supplementary file1 (PDF 2092 kb) [file 204_2020_2779_MOESM1_ESM.pdf]

-Supplemental material-

Hepatotoxic pyrrolizidine alkaloids induce DNA damage response in rat liver in a 28 day feeding study

Johanna Ebmeyer[a], Josef Daniel Rasinger[b], Jan G. Hengstler[c], Dirk Schaudien[d], Otto Creutzenberg[d], Alfonso Lampen[a], Albert Braeuning[a]*, Stefanie Hessel-Pras[a]*

* equally contributed

[a] German Federal Institute for Risk Assessment, Max-Dohrn-Str. 8-10, 10589 Berlin, Germany

[b] Institute of Marine Research (IMR), Postboks 1870 Nordnes, NO-5817 Bergen, Norway

[c] Leibniz Research Centre for Working Environment and Human Factors, Technical University Dortmund, Ardeystr. 67, 44139 Dortmund, Germany

[d] Fraunhofer Institute for Toxicology and Experimental Medicine ITEM, Nikolai-Fuchs-Straße 1, 30625 Hanover, Germany

Corresponding author:
Dr. Stefanie Hessel-Pras
ORC-ID: 0000-0002-6153-0035
German Federal Institute for Risk Assessment
Max-Dohrn-Str. 8-10
10589 Berlin, Germany
E-mail: stefanie.hessel-pras@bfr.bund.de
Tel.: +49-30-18412-25110

Table S1: Subset of 284 probe sets that are significantly different expressed among the treatment groups (one-way ANOVA, q<0.05). Probe sets that are not significantly dysregulated compared to vehicle control as analyzed by post hoc-test (q<0.05) are marked in gray.

fold change

3

0.33

gene symbol	echimidine	heliotrine	lasiocarpine	senecionine	senkirkine	platyphylline	entrez gene name
	**fold change**						
Cenpw	1.16	1.17	1.15	1.77	1.31	1.03	centromere protein W
Lig1	1.09	1.08	1.03	1.23	1.15	1.03	DNA ligase 1
Lig1	1.09	1.10	1.02	1.25	1.16	1.02	DNA ligase 1
Ethe1	0.77	0.83	0.99	0.66	0.76	0.97	ETHE1, persulfide dioxygenase
Aen	1.22	1.41	1.28	1.26	1.30	1.00	apoptosis enhancing nuclease
Ticrr	1.17	1.11	1.07	1.36	1.24	1.01	TOPBP1 interacting checkpoint and replication regulator
Prc1	1.20	1.19	1.05	1.49	1.53	1.05	protein regulator of cytokinesis 1
Rrm1	1.18	1.21	1.03	1.52	1.39	1.09	ribonucleotide reductase catalytic subunit M1
Wee1	1.11	1.14	1.10	1.36	1.20	1.18	WEE1 G2 checkpoint kinase
Acsm3	0.79	0.57	0.77	0.79	0.69	0.89	acyl-CoA synthetase medium chain family member 3
Loc100911413	1.20	1.37	1.18	1.49	0.99	1.03	deleted in malignant brain tumors 1 protein-like
Mgmt	1.58	2.00	1.74	1.75	1.73	0.95	O-6-methylguanine-DNA methyltransferase
Sptbn2	1.01	1.06	1.00	1.07	1.00	0.89	spectrin beta, non-erythrocytic 2
Aldh1A1	2.81	5.20	3.28	3.61	2.84	0.98	aldehyde dehydrogenase 1 family member A1
Fas	1.40	1.64	1.43	1.65	1.42	0.92	Fas cell surface death receptor
Ifit2	1.55	1.29	1.25	1.49	1.42	1.14	interferon induced protein with tetratricopeptide repeats 2
Kif11	1.20	1.35	1.14	1.97	1.67	1.14	kinesin family member 11
Loc100910701	1.02	1.05	0.93	0.95	0.91	0.98	uncharacterized LOC100910701
Fbxo5	1.26	1.06	1.22	1.39	1.39	1.09	F-box protein 5
Axl	0.81	0.80	0.62	0.76	0.85	0.96	AXL receptor tyrosine kinase
Fxyd5	1.19	1.18	1.09	1.19	1.26	0.98	FXYD domain containing ion transport regulator 5
Bax	1.18	1.25	1.21	1.23	1.26	0.98	BCL2 associated X, apoptosis regulator
E2f8	1.20	1.24	1.17	1.45	1.36	1.00	E2F transcription factor 8

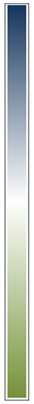

Blm	1.09	1.00	0.98	1.09	1.13	0.96	bloom syndrome RecQ like helicase
Ddias	1.33	1.48	1.31	2.08	1.59	1.15	DNA damage induced apoptosis suppressor
Lyve1	1.25	1.18	1.18	1.19	1.50	1.05	lymphatic vessel endothelial hyaluronan receptor 1
Nupr1	1.26	1.13	1.73	0.97	1.17	1.24	nuclear protein 1, transcriptional regulator
Prss53	1.11	1.04	1.09	0.91	1.08	1.07	protease, serine 53
Mki67	1.50	1.72	1.16	2.68	2.71	1.10	marker of proliferation Ki-67
Adam8	1.83	5.83	1.54	3.76	2.13	1.00	ADAM metallopeptidase domain 8
Ccnd1	1.02	1.14	0.90	1.47	1.13	0.81	cyclin D1
Aldh1a7	1.29	1.68	1.54	1.62	1.32	0.94	aldehyde dehydrogenase family 1, subfamily A7
Fanci	1.14	1.16	1.04	1.44	1.36	1.06	Fanconi anemia complementation group I
Cd81	1.09	1.21	1.15	1.04	1.17	1.08	CD81 molecule
Arsb	1.14	1.30	1.17	1.26	1.20	1.05	arylsulfatase B
Dab2	1.15	1.04	0.98	1.04	1.44	0.99	DAB2, clathrin adaptor protein
Loc102554389	1.18	0.94	1.00	1.10	1.01	1.24	zinc finger protein 862-like
Plk4	1.27	1.22	1.08	1.68	1.41	1.06	Polo like kinase 4
Smc4	1.10	1.24	0.97	1.63	1.46	1.10	structural maintenance of chromosomes 4
Pdgfc	1.13	1.37	1.21	1.24	1.19	0.82	platelet derived growth factor C
Rab13	1.16	1.22	1.11	1.19	1.17	0.95	RAB13, member RAS oncogene family
Hist1h3g	1.32	1.38	1.19	1.84	1.84	1.17	histone cluster 1 H3 family member g
Phtf1	0.84	0.88	0.83	0.88	0.82	0.91	putative homeodomain transcription factor 1
Pde5a	1.07	1.10	0.95	1.25	1.07	0.95	phosphodiesterase 5A
Etnppl	0.91	0.93	1.26	0.71	0.93	1.30	ethanolamine-phosphate phospho-lyase
Cenpe	1.19	1.21	1.06	1.43	1.49	1.01	centromere protein E
Adgrl4	1.23	1.07	1.07	1.12	1.54	0.97	adhesion G protein-coupled receptor L4
Cenph	1.06	1.11	1.07	1.24	1.13	1.05	centromere protein H
Gzma	0.70	0.80	0.63	0.58	0.56	0.87	granzyme A
Lrrcc1	1.24	1.15	1.05	1.44	1.38	1.03	leucine rich repeat and coiled-coil centrosomal protein 1
Ccna2	1.26	1.37	1.09	2.13	1.96	1.13	cyclin A2
Igsf10	1.02	0.92	0.93	1.11	1.08	0.92	immunoglobulin superfamily member 10
Csf1	1.15	1.02	1.00	1.20	1.20	0.96	colony stimulating factor 1
Rgd1310209	1.06	1.12	1.22	0.83	1.12	1.17	similar to KIAA1324 protein

fold change

3

0.33

Loc102548573	0.93	1.02	1.04	1.21	0.93	0.84	uncharacterized LOC102548573
Loc102548789	1.12	1.01	0.99	1.12	0.96	0.98	uncharacterized LOC102548789
Map3k20	1.16	1.23	1.16	1.22	1.15	1.01	mitogen-activated protein kinase kinase kinase 20
Slc43a1	0.87	0.80	0.81	0.76	0.74	0.94	solute carrier family 43 member 1
Kif18a	1.15	1.21	1.07	1.52	1.46	1.04	kinesin family member 18A
Ccdc34	1.07	1.17	1.03	1.32	1.20	1.02	coiled-coil domain containing 34
Knl1	1.26	1.50	1.07	2.07	1.93	1.07	kinetochore scaffold 1
Rad51	1.18	1.25	1.17	1.44	1.34	1.08	RAD51 recombinase
Nusap1	1.23	1.33	1.10	1.66	1.68	1.19	nucleolar and spindle associated protein 1
Loc100911204	1.14	1.37	1.03	1.85	1.70	1.02	protein CASC5-like
Loc100911267	1.14	1.32	1.17	1.59	1.42	1.05	DNA repair protein RAD51 homolog 1-like
Dut	1.18	1.31	1.13	1.37	1.26	1.02	deoxyuridine triphosphatase
Macrod2	0.95	0.96	1.00	0.90	0.96	1.02	MACRO domain containing 2
Tpx2	1.21	1.30	1.07	1.66	1.56	1.07	TPX2, microtubule nucleation factor
Lbp	1.19	1.07	1.07	1.55	1.14	1.10	lipopolysaccharide binding protein
Mybl2	1.35	1.72	1.48	1.65	1.38	1.00	MYB proto-oncogene like 2
Ube2C	1.12	1.16	1.04	1.33	1.29	1.04	ubiquitin conjugating enzyme E2 C
Ntsr1	0.85	0.83	0.79	0.84	0.90	1.17	neurotensin receptor 1
Fcna	0.74	0.69	0.66	0.71	0.74	0.94	ficolin A
Rpl35	1.10	1.03	1.09	0.99	1.06	1.10	ribosomal protein L35
Rbm43	1.11	1.07	1.06	1.17	1.08	0.98	RNA binding motif protein 43
Rcn1	1.20	1.11	1.04	1.25	1.33	0.97	reticulocalbin 1
Arhgap11a	1.23	1.40	1.00	1.67	1.86	1.13	Rho GTPase activating protein 11A
Myef2	1.08	1.07	0.99	1.25	1.16	0.97	myelin expression factor 2
Sppl2a	1.01	1.05	0.94	1.11	1.07	1.10	Ssgnal peptide peptidase like 2A
Ncaph	1.20	1.33	1.12	1.53	1.43	1.07	non-SMC condensin I complex, subunit H
Ckap2l	1.12	1.05	1.03	1.17	1.19	0.97	cytoskeleton associated protein 2 like
Rrbp1	0.94	0.94	0.87	0.96	0.95	0.85	ribosome binding protein 1
E2f1	1.06	1.11	1.10	1.23	1.07	1.02	E2F transcription factor 1
Slpi (Includes Others)	0.95	0.91	0.66	0.72	0.84	0.95	secretory leukocyte peptidase inhibitor
Pltp	0.74	0.68	0.56	0.68	0.79	0.99	phospholipid transfer protein

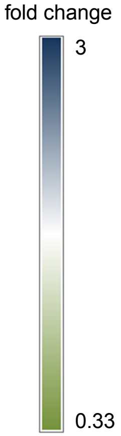

Rbm33	1.00	1.06	0.89	1.00	1.06	0.99	RNA binding motif protein 33
Cav2	1.10	1.00	0.98	1.31	1.08	0.92	caveolin 2
Arf5	1.05	0.99	1.12	0.89	0.97	1.05	ADP ribosylation factor 5
Exoc4	1.05	1.16	1.02	1.19	1.23	0.96	exocyst complex component 4
Akr1b10	1.14	1.05	1.12	1.08	1.46	0.97	aldo-keto reductase family 1 member B10
Mad2l1	1.08	1.13	0.96	1.49	1.22	1.01	mitotic arrest deficient 2 like 1
Uroc1	0.96	0.99	1.04	0.87	0.98	1.10	urocanate hydratase 1
Fancd2	1.13	1.16	1.00	1.49	1.32	0.99	Fanconi anemia complementation group D2
Emp1	1.06	0.98	1.02	1.09	1.31	1.03	epithelial membrane protein 1
Nos3	1.03	0.97	0.98	0.96	1.10	0.94	nitric oxide synthase 3
Abcb1b	2.62	6.75	3.36	5.92	3.33	0.76	ATP-binding cassette, sub-family B (MDR/TAP), member 1B
Abcb1	1.26	1.35	1.22	1.53	1.24	0.94	ATP binding cassette subfamily B member 1
Mkrn1	0.89	0.90	0.88	0.90	0.85	0.93	makorin ring finger protein 1
Inmt	0.63	0.52	0.94	0.43	0.62	0.86	indolethylamine N-methyltransferase
Herc6	1.10	1.26	0.91	1.61	1.22	0.99	HECT and RLD domain containing E3 ubiquitin protein ligase family member 6
Celc4f	0.66	0.59	0.46	0.68	0.63	0.85	C-type lectin domain family 4 member F
Rad18	1.05	1.12	1.04	1.24	1.18	1.06	RAD18, E3 ubiquitin protein ligase
C1s	1.03	1.06	0.92	1.20	1.01	0.97	complement C1s
Klra5 (Includes Others)	1.08	1.12	1.05	1.07	1.12	0.96	killer cell lectin-like receptor, subfamily A, member 5
Klra2	1.27	1.32	1.10	1.35	1.26	0.96	killer cell lectin-like receptor, subfamily A, member 2
Klrc2/Klrc3	0.82	0.89	0.74	0.78	0.80	0.91	killer cell lectin-like receptor subfamily C, member 2
Mybl1	1.66	2.81	1.58	3.67	2.07	0.82	MYB proto-oncogene like 1
Ccne2	1.21	1.30	1.14	1.86	1.54	1.16	cyclin E2
Pip4p2	1.07	1.23	1.07	1.21	1.12	0.99	phosphatidylinositol-4,5-bisphosphate 4-phosphatase 2
Melk	1.11	1.13	1.08	1.29	1.24	1.02	maternal embryonic leucine zipper kinase
Smc2	1.08	1.24	0.97	1.70	1.48	1.08	structural maintenance of chromosomes 2
Stil	1.17	1.23	1.07	1.54	1.45	1.02	STIL, centriolar assembly protein
Clspn	1.12	1.16	1.10	1.27	1.22	1.04	claspin
Stmn1	1.25	1.29	1.10	1.55	1.50	1.03	stathmin 1
Sdhb	1.04	0.97	1.05	0.92	0.96	0.99	succinate dehydrogenase complex iron sulfur subunit B

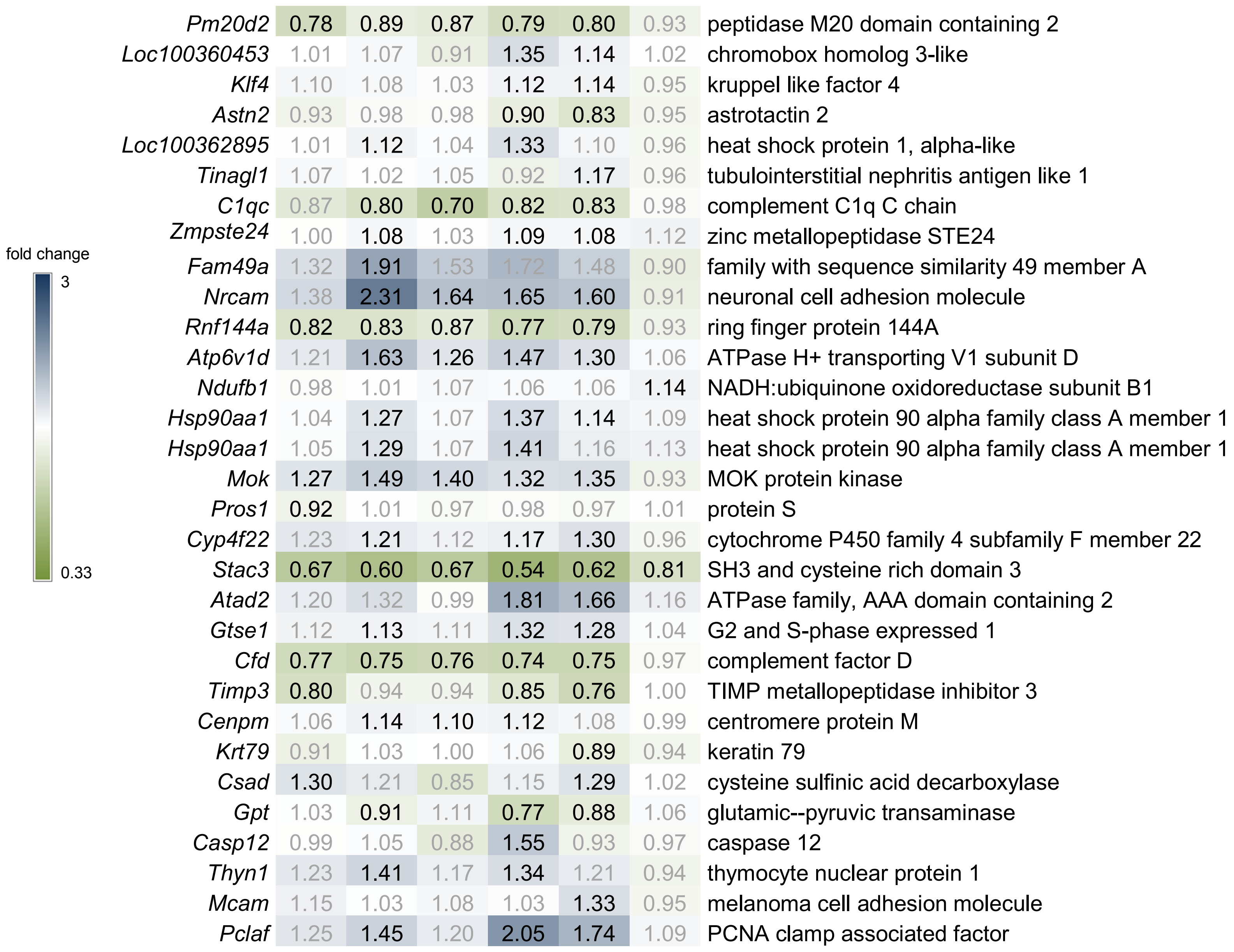
fold change
3
0.33
Pm20d2 0.78 0.89 0.87 0.79 0.80 0.93 peptidase M20 domain containing 2
Loc100360453 1.01 1.07 0.91 1.35 1.14 1.02 chromobox homolog 3-like
Klf4 1.10 1.08 1.03 1.12 1.14 0.95 kruppel like factor 4
Astn2 0.93 0.98 0.98 0.90 0.83 0.95 astrotactin 2
Loc100362895 1.01 1.12 1.04 1.33 1.10 0.96 heat shock protein 1, alpha-like
Tinagl1 1.07 1.02 1.05 0.92 1.17 0.96 tubulointerstitial nephritis antigen like 1
C1qc 0.87 0.80 0.70 0.82 0.83 0.98 complement C1q C chain
Zmpste24 1.00 1.08 1.03 1.09 1.08 1.12 zinc metallopeptidase STE24
Fam49a 1.32 1.91 1.53 1.72 1.48 0.90 family with sequence similarity 49 member A
Nrcam 1.38 2.31 1.64 1.65 1.60 0.91 neuronal cell adhesion molecule
Rnf144a 0.82 0.83 0.87 0.77 0.79 0.93 ring finger protein 144A
Atp6v1d 1.21 1.63 1.26 1.47 1.30 1.06 ATPase H+ transporting V1 subunit D
Ndufb1 0.98 1.01 1.07 1.06 1.06 1.14 NADH:ubiquinone oxidoreductase subunit B1
Hsp90aa1 1.04 1.27 1.07 1.37 1.14 1.09 heat shock protein 90 alpha family class A member 1
Hsp90aa1 1.05 1.29 1.07 1.41 1.16 1.13 heat shock protein 90 alpha family class A member 1
Mok 1.27 1.49 1.40 1.32 1.35 0.93 MOK protein kinase
Pros1 0.92 1.01 0.97 0.98 0.97 1.01 protein S
Cyp4f22 1.23 1.21 1.12 1.17 1.30 0.96 cytochrome P450 family 4 subfamily F member 22
Stac3 0.67 0.60 0.67 0.54 0.62 0.81 SH3 and cysteine rich domain 3
Atad2 1.20 1.32 0.99 1.81 1.66 1.16 ATPase family, AAA domain containing 2
Gtse1 1.12 1.13 1.11 1.32 1.28 1.04 G2 and S-phase expressed 1
Cfd 0.77 0.75 0.76 0.74 0.75 0.97 complement factor D
Timp3 0.80 0.94 0.94 0.85 0.76 1.00 TIMP metallopeptidase inhibitor 3
Cenpm 1.06 1.14 1.10 1.12 1.08 0.99 centromere protein M
Krt79 0.91 1.03 1.00 1.06 0.89 0.94 keratin 79
Csad 1.30 1.21 0.85 1.15 1.29 1.02 cysteine sulfinic acid decarboxylase
Gpt 1.03 0.91 1.11 0.77 0.88 1.06 glutamic--pyruvic transaminase
Casp12 0.99 1.05 0.88 1.55 0.93 0.97 caspase 12
Thyn1 1.23 1.41 1.17 1.34 1.21 0.94 thymocyte nuclear protein 1
Mcam 1.15 1.03 1.08 1.03 1.33 0.95 melanoma cell adhesion molecule
Pclaf 1.25 1.45 1.20 2.05 1.74 1.09 PCNA clamp associated factor

fold change

3

0.33

Anxa2	1.30	1.12	1.09	1.32	1.44	1.02	annexin A2
Ttk	1.19	1.35	1.07	1.85	1.73	1.10	TTK protein kinase
Topbp1	1.11	1.12	0.99	1.28	1.19	1.06	DNA topoisomerase II binding protein 1
Kif15	1.15	1.23	1.11	1.31	1.35	1.02	kinesin family member 15
Cep295	1.08	1.10	1.03	1.12	1.17	0.98	centrosomal protein 295
Spc24	1.04	1.06	1.03	1.13	1.16	0.99	SPC24, NDC80 kinetochore complex component
Anln	1.09	1.14	1.04	1.32	1.31	1.05	anillin actin binding protein
Jam3	1.34	1.87	1.49	1.64	1.43	0.98	junctional adhesion molecule 3
Tbcel	0.97	0.95	0.91	1.09	0.95	1.03	tubulin folding cofactor E like
Kif23	1.10	1.12	1.09	1.26	1.27	1.05	kinesin family member 23
Zwilch	1.30	1.33	1.16	1.76	1.69	1.20	zwilch kinetochore protein
Loc102546846	1.25	1.03	1.00	0.99	1.01	0.98	uncharacterized LOC102546846
Acpp	0.77	0.77	0.91	0.68	0.83	0.87	acid phosphatase, prostate
Cmtm8	1.17	1.11	1.23	1.07	1.10	1.04	CKLF like MARVEL transmembrane domain containing 8
Gnmt/Loc100911564	0.81	0.84	0.94	0.69	0.75	0.89	glycine N-methyltransferase
Gnmt/Loc100911564	0.80	0.83	0.94	0.68	0.74	0.88	glycine N-methyltransferase
Klc4	1.01	0.96	1.00	0.89	0.95	1.00	kinesin light chain 4
Cenpq	1.20	1.32	1.16	1.79	1.43	1.10	centromere protein Q
Map4K4	1.08	1.03	0.94	1.12	1.20	0.91	mitogen-activated protein kinase kinase kinase kinase 4
Inpp1	1.18	1.21	1.07	1.35	1.11	0.96	inositol polyphosphate-1-phosphatase
Aox4	0.84	0.86	0.78	0.79	0.82	0.94	aldehyde oxidase 4
Cd28	1.13	1.09	1.09	1.04	1.42	1.09	CD28 molecule
Alkbh7	1.05	1.05	1.05	0.90	0.96	1.07	AlkB homolog 7
Uhrf1	1.18	1.18	1.16	1.33	1.34	1.03	ubiquitin like with PHD and ring finger domains 1
Mcm3	1.13	1.31	1.09	1.54	1.34	1.09	minichromosome maintenance complex component 3
Gsta1	1.17	1.31	1.28	1.21	1.25	1.13	glutathione S-transferase alpha 1
Gsta1	1.19	1.38	1.32	1.29	1.29	1.16	glutathione S-transferase alpha 1
Nhej1	1.12	1.18	1.21	1.11	1.13	0.92	non-homologous end joining factor 1
Hjurp	1.17	1.10	1.07	1.25	1.17	1.00	holliday junction recognition protein
Ndc80	1.25	1.37	1.06	1.79	1.72	1.12	NDC80, kinetochore complex component
Tyms	1.32	1.61	1.30	1.82	1.65	1.09	thymidylate synthetase

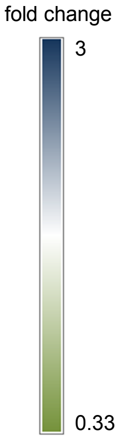

Heph	1.21	1.28	1.09	1.14	1.14	1.01	hephaestin
Kif4A	1.17	1.21	1.04	1.40	1.44	1.02	kinesin family member 4A
Gria3	1.45	1.99	1.46	1.55	1.69	1.00	glutamate ionotropic receptor AMPA type subunit 3
Loc100912524/Rgd1565862	0.98	0.87	1.03	0.79	0.94	1.03	similar to Spindlin-like protein 2 (SPIN-2)
Eda2r	2.40	4.78	2.80	3.54	4.18	1.00	ectodysplasin A2 receptor
Ercc6l	1.24	1.25	1.40	1.39	1.49	1.21	ERCC excision repair 6 like, spindle assembly checkpoint helicase
Il13ra2	1.31	1.13	1.01	1.31	1.52	0.99	interleukin 13 receptor subunit alpha 2
Mpv17	0.95	0.96	1.02	0.98	0.91	1.10	MPV17, mitochondrial inner membrane protein
Adam19	1.06	1.03	1.05	1.02	1.24	0.97	ADAM metallopeptidase domain 19
Aurkb	1.14	1.19	1.11	1.45	1.31	1.06	aurora kinase B
Ankfy1	0.96	0.95	0.86	1.06	1.04	0.96	ankyrin repeat and FYVE domain containing 1
Myo1c	1.04	0.98	0.97	1.01	1.13	0.96	myosin IC
Atad5	1.08	1.15	1.07	1.34	1.25	0.99	ATPase family, AAA domain containing 5
Rpl19	1.16	1.21	1.19	1.19	1.16	1.07	ribosomal protein L19
Hmmr	1.16	1.41	1.00	2.14	1.75	1.16	hyaluronan mediated motility receptor
Ccng1	1.43	2.04	1.61	1.91	1.59	0.99	cyclin G1
Gabrg2	1.11	1.02	1.19	1.03	0.98	1.07	gamma-aminobutyric acid type A receptor gamma2 subunit
Loc103693330/Loc103693430	1.08	1.05	1.11	1.01	1.08	1.24	up-regulated during skeletal muscle growth protein 5-like
Tmem88	1.11	1.01	1.14	1.05	1.36	0.98	transmembrane protein 88
Shbg	1.05	0.99	1.06	0.89	1.00	0.98	sex hormone binding globulin
Chrne	1.27	1.15	1.16	0.95	1.18	1.09	cholinergic receptor nicotinic epsilon subunit
Sez6	0.88	0.76	0.86	0.76	0.86	0.86	seizure related 6 homolog
Slfn13	1.05	0.98	0.86	1.01	1.13	0.95	schlafen family member 13
Top2a	1.30	1.52	1.00	2.64	2.35	1.16	DNA topoisomerase II alpha
Brca1	1.16	1.12	0.97	1.39	1.32	1.02	BRCA1, DNA repair associated
Lgals3bp	1.24	1.44	1.05	1.38	1.42	0.97	galectin 3 binding protein
Spag5	1.05	1.10	0.98	1.21	1.17	0.98	sperm associated antigen 5
Olfr209	1.00	1.09	1.12	1.20	1.05	0.96	olfactory receptor 209
Hmgb2 (Includes Others)	1.21	1.25	1.11	1.46	1.42	0.99	high mobility group box 2
Gcsam	0.78	0.91	0.72	0.72	0.77	0.89	germinal center associated signaling and motility
Cd80	1.16	1.11	1.11	1.26	1.51	0.96	CD80 molecule

fold change

3

0.33

Golgb1	0.86	0.87	0.84	0.93	0.96	0.85	golgin B1
Loc102553356	1.77	1.57	1.58	1.13	1.70	1.13	uncharacterized LOC102553356
Loc680825	0.81	0.74	0.73	0.74	0.79	0.93	hypothetical protein LOC680825
Kntc1	1.18	1.30	1.11	1.54	1.51	1.07	kinetochore associated 1
Oasl2	1.10	1.16	0.89	1.16	1.17	0.95	2'-5' oligoadenylate synthetase-like 2
Acmsd	0.72	0.76	0.93	0.99	0.89	1.16	aminocarboxymuconate semialdehyde decarboxylase
Loc102555796	1.13	1.14	1.18	1.04	1.01	1.13	uncharacterized LOC102555796
Kdm5b	0.83	0.86	0.78	0.85	0.88	0.84	lysine demethylase 5B
Ube2t	1.11	1.20	1.12	1.21	1.23	1.07	ubiquitin conjugating enzyme E2 T
Kif14	1.07	1.02	1.03	1.16	1.10	0.99	kinesin family member 14
Sh2d1b	1.06	1.29	0.99	1.25	1.16	1.02	SH2 domain containing 1B
Cd244	1.01	1.09	0.90	1.02	0.98	0.90	CD244 molecule
Exo1	1.06	1.11	1.05	1.15	1.19	1.00	exonuclease 1
Loc690147	1.19	1.43	1.18	1.34	1.29	0.93	similar to Epoxide hydrolase 1 (Microsomal epoxide hydrolase) (Epoxide hydratase)
Kiss1	1.13	1.05	1.18	0.96	1.06	1.05	KiSS-1 metastasis-suppressor
Slamf7	0.87	0.96	0.82	0.83	0.93	1.00	SLAM family member 7
Ephx1	1.21	1.51	1.29	1.39	1.39	1.01	epoxide hydrolase 1
Cenpf	1.06	1.08	0.95	1.30	1.27	0.96	centromere protein F
Dtl	1.23	1.32	1.16	1.67	1.54	1.05	denticleless E3 ubiquitin protein ligase homolog
Cr1l	1.19	1.43	1.29	1.32	1.27	1.02	complement C3b/C4b receptor 1 like
Gbp6	1.18	1.29	0.92	1.46	1.23	1.03	guanylate binding protein family member 6
Hsd17b13	0.95	0.97	1.04	0.88	0.95	1.01	hydroxysteroid 17-beta dehydrogenase 11
Hsd17b13	1.33	1.49	1.52	1.21	1.35	1.27	hydroxysteroid 17-beta dehydrogenase 13
Ugt2b17	1.40	1.34	1.69	1.05	1.33	1.11	UDP glucuronosyltransferase family 2 member B17
Sgcb	1.16	1.55	1.27	1.53	1.38	1.05	sarcoglycan beta
Qdpr	0.98	0.89	1.01	0.86	0.90	1.02	quinoid dihydropteridine reductase
Man2b2	1.10	1.14	1.02	1.15	1.10	0.95	mannosidase alpha class 2B member 2
Bmp3	1.12	1.06	1.11	1.03	1.64	1.01	bone morphogenetic protein 3
Dck	1.07	1.19	1.04	1.36	1.27	1.06	deoxycytidine kinase
Kit	1.13	1.08	1.05	1.00	1.43	0.96	KIT proto-oncogene receptor tyrosine kinase

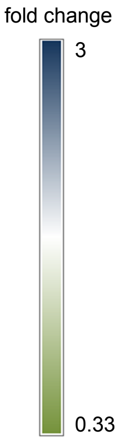

Tacc3	1.12	1.12	1.02	1.28	1.21	1.04	transforming acidic coiled-coil containing protein 3
Loxl2	1.09	0.99	0.98	1.05	1.24	0.94	lysyl oxidase like 2
Pcdh17	1.19	1.03	1.06	1.14	1.44	0.99	protocadherin 17
Wdhd1	1.07	1.11	0.92	1.14	1.17	0.97	WD repeat and HMG-box DNA binding protein 1
Dlgap5	1.14	1.12	1.00	1.54	1.32	1.01	DLG associated protein 5
Ndrg2	0.94	0.94	0.97	0.86	0.92	1.00	NDRG family member 2
Ebpl	1.14	1.12	1.19	1.12	1.21	1.16	emopamil binding protein like
Esco2	1.30	1.26	1.13	2.37	1.95	1.16	establishment of sister chromatid cohesion N-acetyltransferase 2
Cdca2	1.15	1.14	1.04	1.35	1.26	1.02	cell division cycle associated 2
Loc102549099	1.38	1.81	1.66	1.54	1.52	1.04	uncharacterized LOC102549099
Diaph3	1.27	1.45	1.11	1.70	1.78	1.04	diaphanous related formin 3
Il17rd	1.31	2.80	1.77	2.00	1.38	0.85	interleukin 17 receptor D
Chdh	1.01	0.93	1.09	0.87	0.98	1.03	choline dehydrogenase
Loc102553008	0.87	0.77	0.87	0.55	0.82	1.10	uncharacterized LOC102553008
Nrg1	1.14	1.70	1.26	1.62	1.20	0.91	neuregulin 1
Ckap2	1.62	2.08	1.25	3.12	2.34	1.14	cytoskeleton associated protein 2
Loc100360260	1.03	1.03	0.94	1.45	1.20	1.03	chromobox homolog 3-like
Hmgb2 (Includes Others)	1.16	1.16	1.12	1.46	1.27	1.06	high mobility group box 2
Hpgd	0.66	0.71	0.67	0.69	0.71	0.94	15-hydroxyprostaglandin dehydrogenase
Dctd	1.13	1.16	0.96	1.29	1.35	1.00	dCMP deaminase
Cenpu	1.13	1.17	1.13	1.43	1.31	1.02	centromere protein U
Hist1h2bb	1.20	1.07	1.07	1.26	1.28	1.13	histone cluster 1 H2B family member b
Mastl	1.43	1.63	1.14	2.57	1.99	1.20	microtubule associated serine/threonine kinase like
Znf367	1.21	1.26	1.15	1.57	1.42	0.97	zinc finger protein 367
Cd14b	1.08	1.07	1.10	1.13	1.16	1.22	cell division cycle 14B
Hist1h2ab	1.12	1.10	1.10	1.28	1.28	1.05	histone cluster 1 H2A family member b
Rbbp8	1.07	1.12	1.06	1.41	1.16	1.02	RB binding protein 8, endonuclease
Csf1r	0.61	0.62	0.46	0.61	0.65	0.92	colony stimulating factor 1 receptor
Tubb6	1.07	1.12	1.06	1.21	1.27	1.03	tubulin beta 6 class V
Slc39a6	1.27	1.27	1.09	1.19	1.27	1.06	solute carrier family 39 member 6
Nr3c1	0.76	0.82	0.80	0.67	0.76	0.99	nuclear receptor subfamily 3 group C member 1

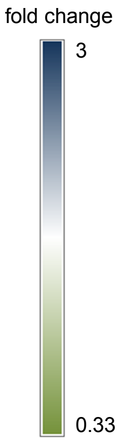

Mcm5	1.29	1.33	1.10	1.72	1.50	1.07	Minichromosome maintenance complex component 5
Cdh13	1.03	1.38	1.09	1.17	1.01	0.96	cadherin 13
Itgb1	1.03	1.07	0.95	1.15	1.13	0.99	integrin subunit beta 1
Coq9	1.02	0.95	1.01	0.93	0.92	0.99	coenzyme Q9
H2-T22	1.14	1.07	1.01	1.00	1.13	0.96	histocompatibility 2, T region locus 22
Tcf19	1.12	1.20	0.98	1.52	1.40	1.03	transcription factor 19
Tap1	1.08	1.16	0.95	1.21	1.06	0.94	transporter 1, ATP binding cassette subfamily B member
Cdkn1a	2.06	3.13	2.44	2.72	2.28	1.13	cyclin dependent kinase inhibitor 1A
Pln	1.13	1.31	1.02	1.44	1.07	1.04	phospholamban
Fyn	1.14	1.13	1.05	1.10	1.23	1.01	FYN proto-oncogene, Src family tyrosine kinase
Hace1	1.00	1.10	1.05	1.20	1.06	0.97	HECT domain and ankyrin repeat containing E3 ubiquitin protein ligase 1
Ftcd	0.96	0.95	0.97	0.83	0.90	1.01	formimidoyltransferase cyclodeaminase
Slc25a16	1.05	1.03	0.99	1.11	1.10	1.00	solute carrier family 25 member 16
Clic1	1.11	1.02	1.02	1.11	1.18	0.95	chloride intracellular channel 1
Fcgr2a	0.86	0.86	0.70	0.86	0.87	0.98	Fc fragment of IgG receptor IIa

Table S2: Summary of probe sets that are significantly different expressed by 3.3 mg individual PA per kg bw compared to vehicle control (one-way ANOVA, q<0.05).

PA	*gene symbol*	entrez gene name
echimidine	*Loc102554389*	zinc finger protein 862-like
	Loc102546846	uncharacterized LOC102546846
	Pros1	protein S
heliotrine	*Tbcel*	tubulin folding cofactor E like
	Pdgfc	platelet derived growth factor C
	Fam49a	family with sequence similarity 49 member A
	Nhej1	non-homologous end joining factor 1
lasiocarpine	*Gabrg2*	gamma-aminobutyric acid type A receptor gamma2 subunit
	Kiss1	KiSS-1 metastasis-suppressor
	Ankfy1	ankyrin repeat and FYVE domain containing 1
	Rrbp1	ribosome binding protein 1
	Cd244	CD244 molecule
	Nupr1	nuclear protein 1, transcriptional regulator
senecionine	*C1s*	complement C1s
	Ccnd1	cyclin D1
	Sppl2a	signal peptide peptidase like 2A
	Uroc1	urocanate hydratase 1
	Hsd17b11	hydroxysteroid 17-beta dehydrogenase 11
	Gbp6	guanylate binding protein family member 6
	Klc4	kinesin light chain 4
	E2f1	E2F transcription factor 1
	Igsf10	immunoglobulin superfamily member 10
	Shbg	sex hormone binding globulin
	Chdh	choline dehydrogenase
	Abcb1b	ATP binding cassette subfamily B member 1
	Sdhb	succinate dehydrogenase complex iron sulfur subunit B
	Herc6	HECT and RLD domain containing E3 ubiquitin protein ligase family member 6
	Cav2	caveolin 2
	Casp12	caspase 12
senkirkine	*Emp1*	epithelial membrane protein 1
	Bmp3	bone morphogenetic protein 3
	Rgd1310209	similar to KIAA1324 protein
	Nos3	nitric oxide synthase 3
	Mcam	melanoma cell adhesion molecule
	Adgrl4	adhesion G protein-coupled receptor L4
	Krt79	keratin 79
	Clic1	chloride intracellular channel 1
	Akr1B10	aldo-keto reductase family 1 member B10
	Tinagl1	tubulointerstitial nephritis antigen like 1
	Loxl2	lysyl oxidase like 2
	Tmem88	transmembrane protein 88
	Cd28	CD28 molecule
	Adam19	ADAM metallopeptidase domain 19
	Kit	KIT proto-oncogene receptor tyrosine kinase
	Dab2	DAB2, clathrin adaptor protein
	Loc100910701	uncharacterized LOC100910701
	Myo1c	myosin 1C
	Mpv17	MPV17, mitochondrial inner membrane protein

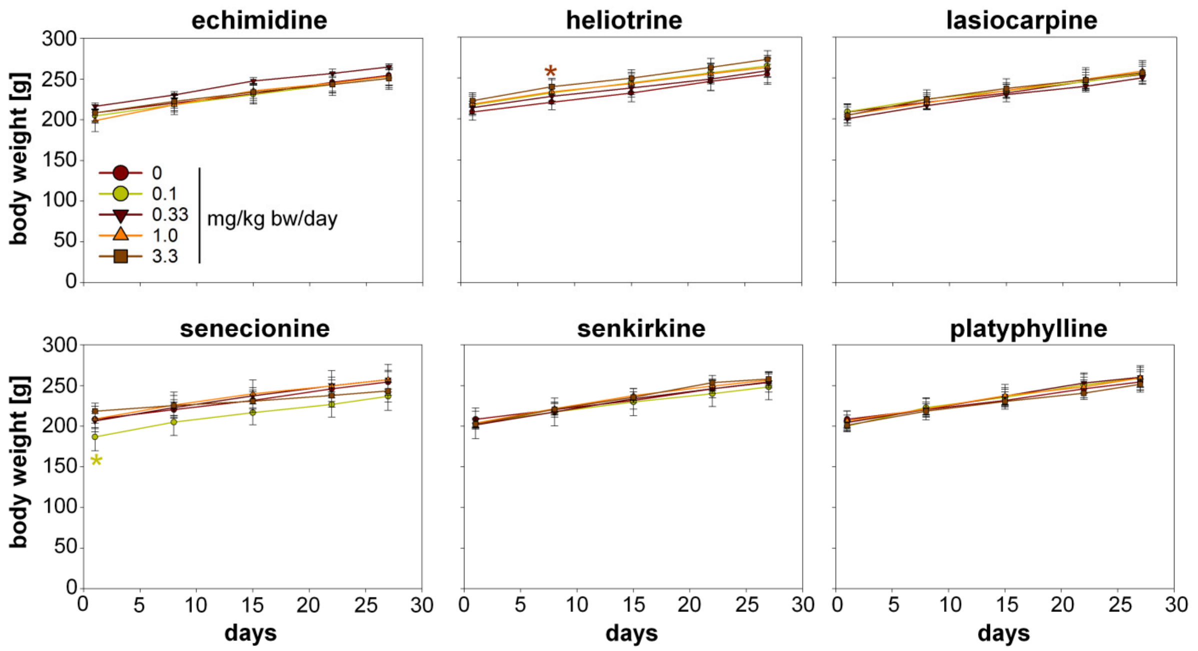

Fig. S1: Development of body weight of all animals throughout the study. Individual body weights were recorded to the nearest 0.1 g once a week on day 1, 8, 15, 22 and 27. Data are shown as mean ± SD of all 5 animals of the same treatment group (8 animals in vehicle group). Statistical significant differences were calculated by one-way ANOVA followed by Dunnett's post-hoc test ($p<0.05$).

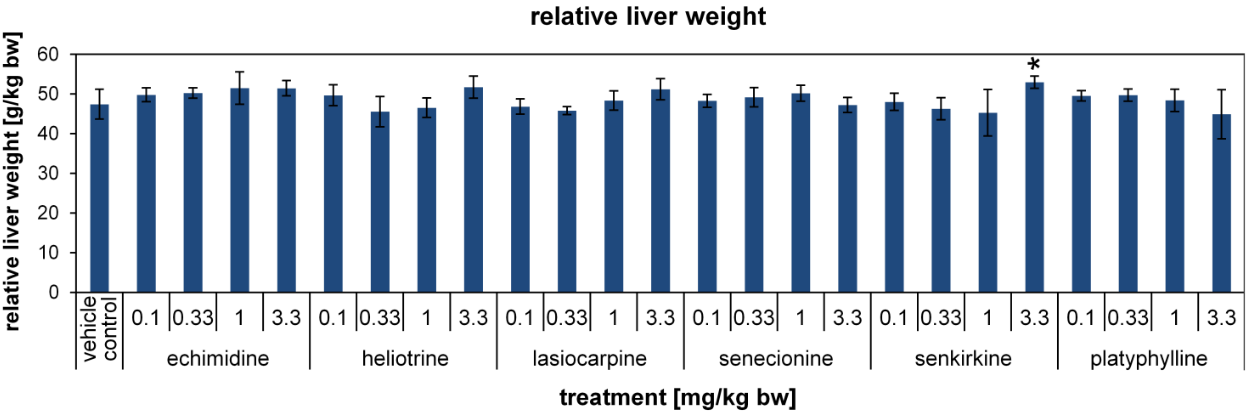

Fig. S2: Relative liver weight for all animals at the end of the study. Wet liver weights were recorded upon necropsy on day 29 (1 day after the last treatment) and related to the body weight upon necropsy. Data are shown as mean ± SD of all 5 animals of the same treatment group (8 animals in vehicle group). Statistical significant differences vs. vehicle control were calculated by one-way ANOVA followed by Dunnett's post-hoc test ($p<0.05$).

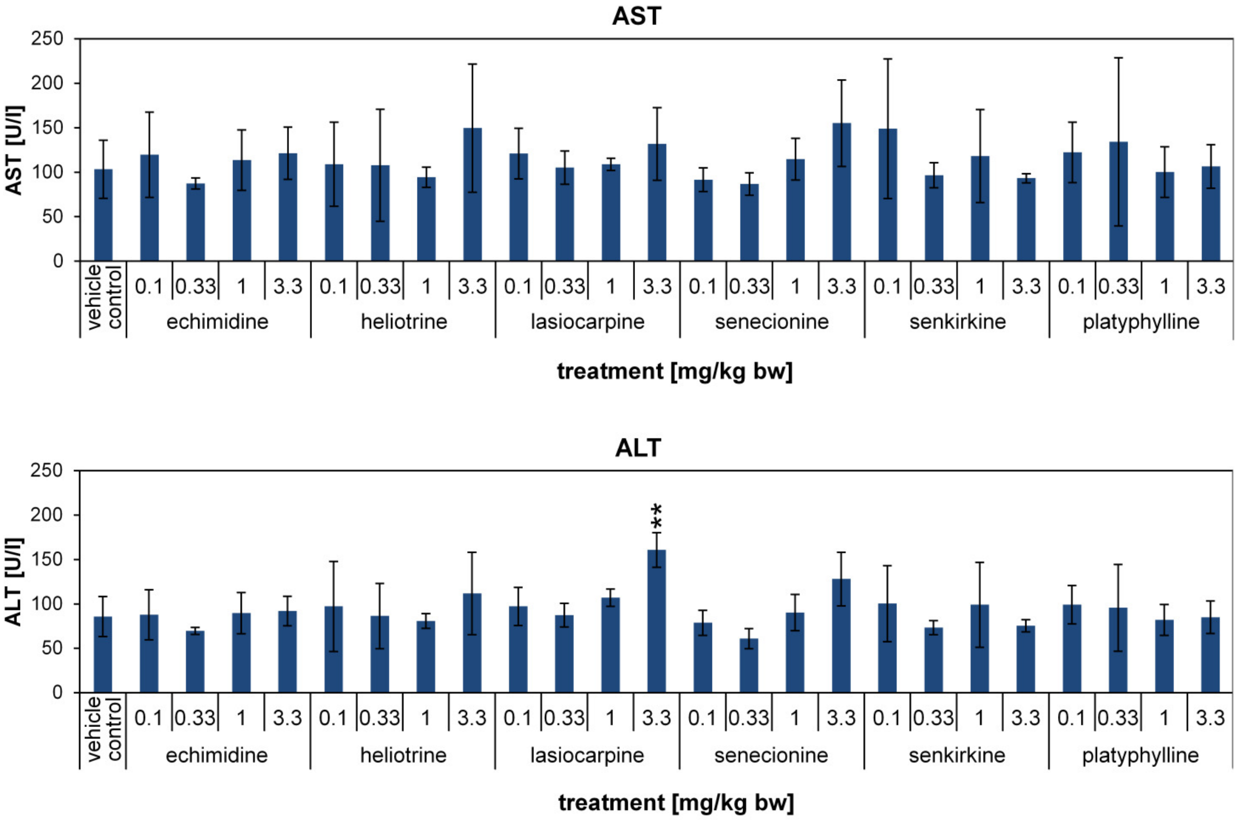

Fig. S3: Levels of the enzymes aspartate aminotransferase (AST) and alanine aminotransferase (ALT) in the blood plasma of the treated animals. AST and ALT levels were analyzed in the blood plasma sampled on day 29. Mean results ± SD of all 5 animals of the same treatment group (8 animals in vehicle group) are shown. Statistical significant differences vs. the vehicle control were analyzed by one-way ANOVA followed by Dunnett's post-hoc test (**p<0.01).

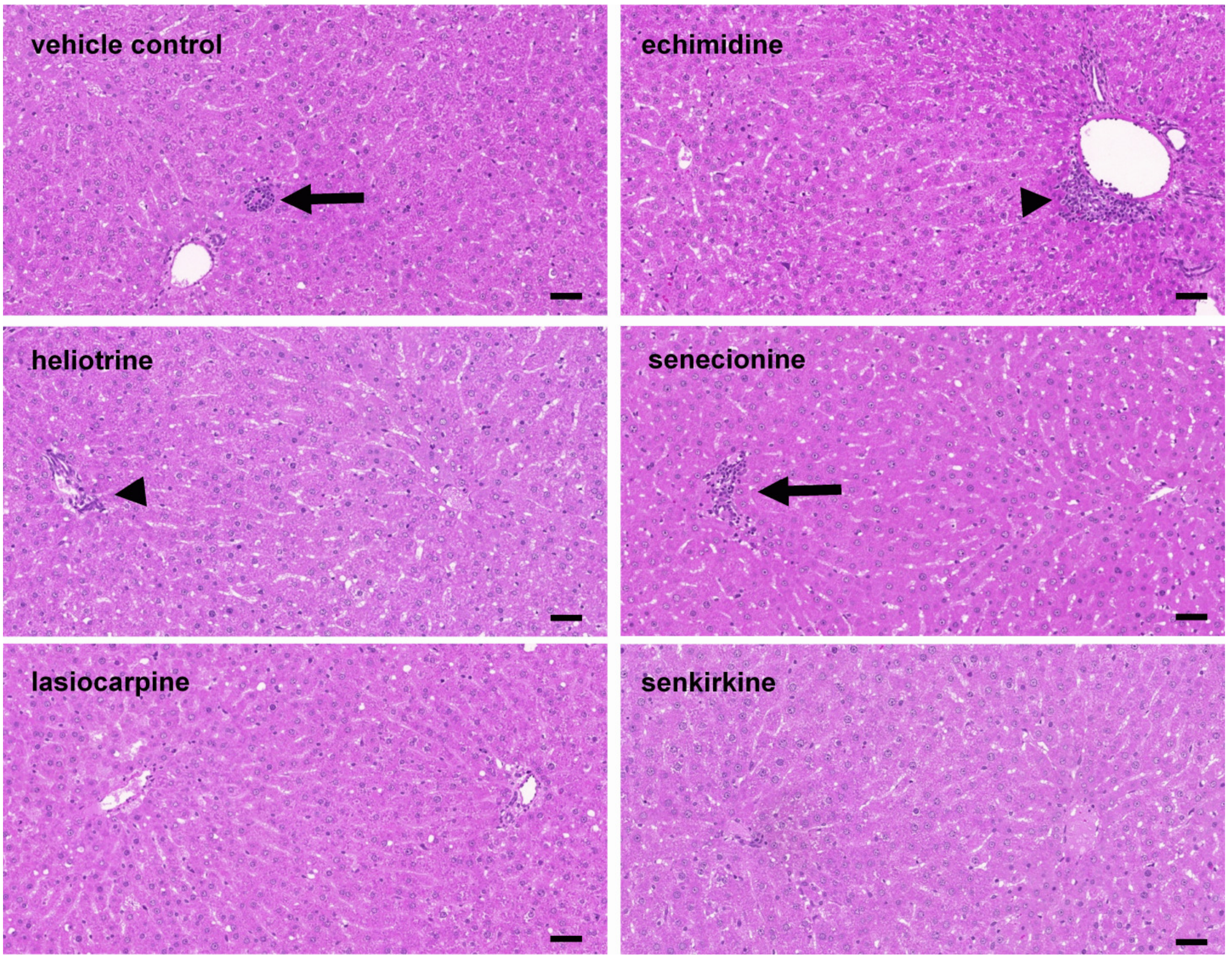

Fig. S4: Histological slides stained with hematoxylin and eosin of the livers of male Fischer rats orally treated daily for 28 days with 3.3 mg/kg body weight of the respective pyrrolizidine alkaloid or vehicle control. A multifocal random mixed inflammatory cell infiltration (arrows) as well as multifocal periportal mononuclear cell infiltration (arrow heads) were seen to a very slight extent in up to two animals per group in most groups. The lesions represent commonly found background lesions in rat livers. Portal or biliary fibrosis, bile duct proliferation, hepatocellular degeneration (necrosis), swelling of hepatocytes, megalocytosis or karyomegaly of liver cells or any other adverse finding were not seen in any group. Bar = 50 µm.

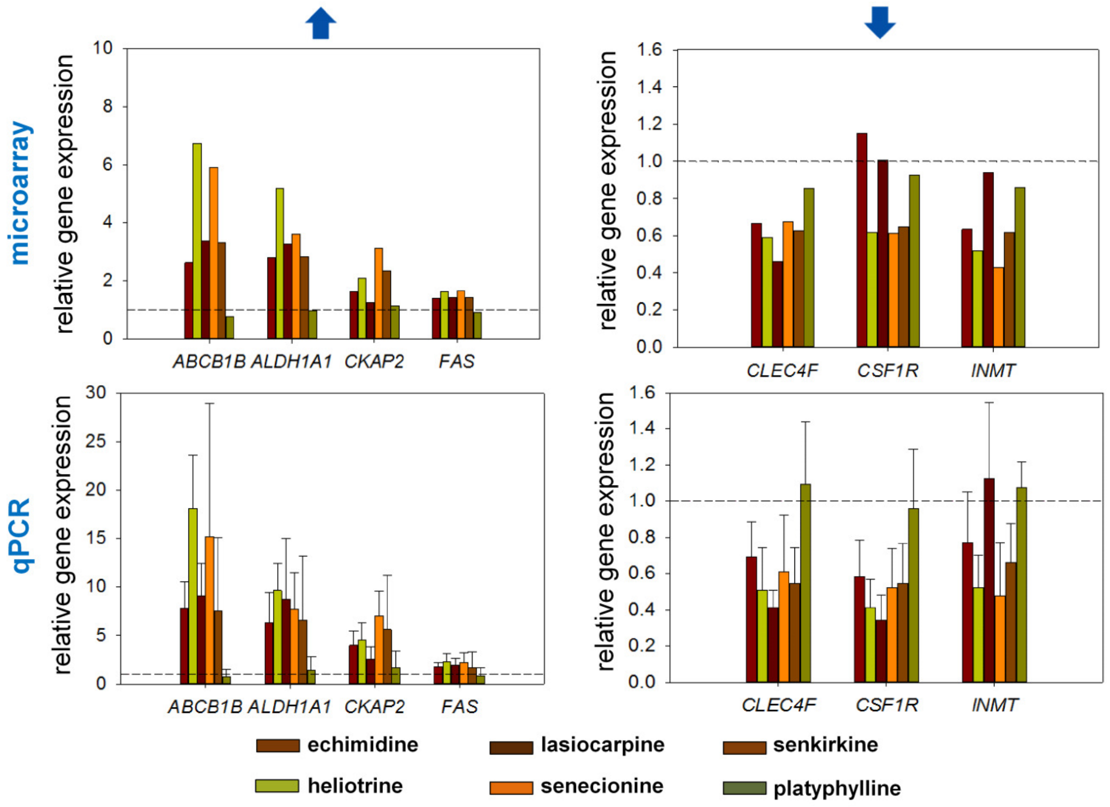

Fig. S5: Validation of PA-induced gene regulation in rat livers by qPCR. Total mRNA of rat liver samples was analyzed by qPCR for the expression of selected genes. A total of in the microarray analysis 4 up- and 3 downregulated genes was analyzed and results from microarray analysis (top) and qPCR (bottom) were compared. Fold changes compared to vehicle control are shown as mean ± SD of 5 individual animals.
